# The Recombinational Anatomy of a Mouse Chromosome

**DOI:** 10.1371/journal.pgen.1000119

**Published:** 2008-07-11

**Authors:** Kenneth Paigen, Jin P. Szatkiewicz, Kathryn Sawyer, Nicole Leahy, Emil D. Parvanov, Siemon H. S. Ng, Joel H. Graber, Karl W. Broman, Petko M. Petkov

**Affiliations:** 1 Center for Genome Dynamics, The Jackson Laboratory, Bar Harbor, Maine, United States of America; 2 Department of Biostatistics and Medical Informatics, University of Wisconsin, Madison, Wisconsin, United States of America; National Cancer Institute, United States of America

## Abstract

Among mammals, genetic recombination occurs at highly delimited sites known as recombination hotspots. They are typically 1–2 kb long and vary as much as a 1,000-fold or more in recombination activity. Although much is known about the molecular details of the recombination process itself, the factors determining the location and relative activity of hotspots are poorly understood. To further our understanding, we have collected and mapped the locations of 5,472 crossover events along mouse Chromosome 1 arising in 6,028 meioses of male and female reciprocal F1 hybrids of C57BL/6J and CAST/EiJ mice. Crossovers were mapped to a minimum resolution of 225 kb, and those in the telomere-proximal 24.7 Mb were further mapped to resolve individual hotspots. Recombination rates were evolutionarily conserved on a regional scale, but not at the local level. There was a clear negative-exponential relationship between the relative activity and abundance of hotspot activity classes, such that a small number of the most active hotspots account for the majority of recombination. Females had 1.2× higher overall recombination than males did, although the sex ratio showed considerable regional variation. Locally, entirely sex-specific hotspots were rare. The initiation of recombination at the most active hotspot was regulated independently on the two parental chromatids, and analysis of reciprocal crosses indicated that parental imprinting has subtle effects on recombination rates. It appears that the regulation of mammalian recombination is a complex, dynamic process involving multiple factors reflecting species, sex, individual variation within species, and the properties of individual hotspots.

## Author Summary

In most eukaryotic organisms, recombination—the exchange of genetic information between homologous chromosomes—ensures the proper recognition and segregation of chromosomes during meiosis. Recombination events in mammals are not randomly positioned along the chromosomes but occur in preferential 1–2-kilobase sequences termed hotspots. Different species such as humans and mice do not share hotspots, although the same principles almost certainly regulate their placement in the genome. Hotspot positions and activities depend on genetic background and show sex-specific differences. In this study, we present a detailed analysis of recombination activity along the largest mouse chromosome, finding that recombination is regulated on multiple levels, including regional positioning relative to the chromosomal ends, local gene content, sex-specific mechanisms of hotspot recognition, and parental origin. Our results will contribute to further understanding of one of the most fundamental biological processes and are likely to cast light on several aspects of population genetics and evolutionary biology, as well as enhance our practical ability to define the genetic components of human disease.

## Introduction

Genetic recombination is a fundamental process common to all eukaryotic organisms, which ensures proper chromosomal segregation of homologous chromosomes in meiosis and increases genetic diversity by creating new combinations of parental alleles at each generation. The process begins in the leptotene stage of meiosis I with the creation of double strand breaks on one chromatid by the topoisomerase-like protein *Spo11*, and is followed by resection of 5′-ends to leave 3′-overhangs which then displace existing strands on a non-sister chromatid. The resected regions are eventually repaired using the non-sister chromatid as a template, producing two types of recombination products: crossovers, and gene conversions without exchange of flanking markers (non-crossovers). According to the most widely accepted model of double-strand break processing [1], crossovers are predominantly produced by double-strand break repair (DSBR), and non-crossovers are predominantly produced by synthesis-dependent strand annealing (SDSA) [2].

In mammals, higher plants and yeast, recombination initiates prior to synapsis, and is required for successful chromosome pairing in meiosis I. The majority of recombination is localized to very limited intervals along the genome, termed hotspots, which in mammals are typically only 1-2 kilobase pairs (kb) long [3] and are surrounded by much longer regions (tens of kilobases or more) lacking recombination. When crossover rates are measured at individual hotspots on sperm samples [4], their activities vary over several orders of magnitude, from as high as 1–2 centimorgans (cM) [5] to below 0.001 cM. In contrast, hotspots are not believed to be present in organisms such as *Drosophila* and *C. elegans* where synapsis precedes recombination [6],[7], although local variation of recombination rates across large genomic regions exists in these organisms [8],[9].

Despite their apparent abundance, less than two dozen recombination hotspots have been experimentally analyzed [10]–[13] in humans and mice. The most intensely mapped mammalian regions are the *H2* region of mouse Chromosome 17 [5],[14], the human *HLA* region of Chromosome 6 [3], and the *Ath1* region of mouse Chr 1 [11]. The evidence emerging from these studies suggests that mammalian hotspots are not uniformly or even randomly located along chromosomes. They can occur in “torrid zones” of very high recombination, with clusters of hotspots within 100 kb [11], leaving long stretches of DNA (as much as a megabase or more) devoid of recombination.

Recombination positioning and activity differ significantly between the sexes, and their recombination maps can have different lengths in many species. The female map is about 1.7 times longer than the male map in humans [15],[16] and about 1.3 times in mice [17], and high-resolution sex-specific linkage maps in humans [18] and mice [17] show dramatic variation between male and female recombination rates along the chromosomes. Several explanations have been proposed for these sex differences, including haploid selection[19], different epistatic interactions for genes expressed paternally or maternally[20], and regional differences in the chromatin structure of male and female gametocytes [21]. Our own work has shown that a difference in crossover interference distances in Mb, related to the physical length of synaptonemal complexes at the pachytene stage of meiosis I [22], is a major factor underlying broad-scale sex differences in recombination rates. Sex specificity has also been detected at the level of individual hotspots [23], resulting from participation of both *cis*- and *trans*-acting factors [5].

In several species, including maize [24], humans [25]–[27] and mice [5],[23], genetic background can dramatically influence the placement and activity of hotspots. Humans and chimpanzees do not share hotspots, although their sequences are 98.6% identical [28],[29]. Differences in recombination activity between individual human males were detected even when the hotspot and its surrounding sequences were identical [27]. And, in perhaps the most extensive such study, the activity of the *Psmb9* hotspot in mice is dependent on flanking sequences, even though the hotspot sequence itself is identical in both active and inactive haplotypes [5],[23].

Collectively, these findings emphasize the utility of defining the recombination landscape resulting from hotspots acting in a genetically defined background, a task that is impossible in humans but entirely feasible in experimental animals. Creating such high-resolution genetic maps is important for both theoretical and practical reasons. Studying one-generation recombination in a genetically defined system will provide an entrée to understanding how the recombination process is regulated, the mechanisms underlying sex specificity, and the role of hotspots in evolutionary processes. Better fine-scale genetic maps will also help optimize strategies for mapping and identifying genes underlying disease that rely on genome-wide association studies in humans and the analysis of quantitative trait loci in laboratory animals.

Several genome-wide mapping efforts in mice [30]–[32] have achieved near centimorgan resolution, the latest and most comprehensive one reaching an average resolution of 0.37 cM or 550 kb [17]. The goal of this study is to present the first detailed analysis of recombination on an entire chromosome of an experimental animal under genetically defined circumstances at a resolution power reaching <5 kb that enables detection of individual hotspots.

## Results

### Mapping Strategy

We studied sex-specific recombination rates along the entirety of mouse Chr 1 as they occurred in the meioses of C57BL/6J (B6) and CAST/EiJ (CAST) F1 hybrids of both sexes at an average resolution of 225 kb, and further refined the extended subtelomeric region of 24.7 Mb. To test for potential effects parental imprinting might have on recombination, the F1 animals were produced by reciprocal crosses, and then backcrossed to C57BL/6J. Mapping the location of crossovers in these backcross progeny provided information on the recombination events arising in the F1 hybrids. A total of 6028 progeny were genotyped, of which 1465 were offspring of female B6xCAST, 1537 of female CASTxB6, 1479 of male B6xCAST, and 1547 of male CASTxB6. In all, we detected and localized 5472 crossover events on Chr 1, reaching a genetic resolution of 0.017 cM in the combined offspring. The frequency with which chromosomes with different numbers of crossovers were observed is summarized in Table 1. We found significantly more multiple crossovers in female compared to male meiosis (p<10^−13^ by χ^2^ test) as described before [22].

**Table 1:** Distribution of crossovers on Chr 1.

Number of Crossovers per Chromosome	0	1	2	3	4	Total Samples Tested
Female B6xCAST	363	750	331	19	2	1465
Female CASTxB6	432	735	342	28	0	1537
**Total Female**	**795**	**1485**	**673**	**47**	**2**	**3002**
Male B6xCAST	517	731	226	5	0	1479
Male CASTxB6	516	770	259	2	0	1547
**Total Male**	**1033**	**1501**	**485**	**7**	**0**	**3026**

Backcross offspring were genotyped in two consecutive rounds with single nucleotide polymorphism (SNP) assays developed using the Amplifluor system (see Materials and Methods). In the first round, all progeny DNAs were mapped over the entire chromosome at 10-Mb resolution. This was sufficient to detect virtually all crossovers, given the strong interference in mouse meiosis [33]. In the second round, the crossovers occurring in each interval were mapped using additional SNP markers to an average physical resolution of 225 Kb. To provide a sample of even more detailed information, recombinants in the subtelomeric 24.7 Mb were subjected to additional rounds of testing using a combination of SNP and simple sequence length polymorphism (SSLP) markers. Among the crossovers occurring in this region, 81.4% were mapped to under 100 kb resolution: 8.2% at 50–100 kb resolution, 33.5% at 20–50 kb resolution, 8.6% to a nearly hotspot resolution of 5–20 kb and 31.1% were mapped to <5 kb, ensuring hotspot level resolution. All markers used in this study, their positions according to NCBI Build 36, physical resolution and the number of crossovers in each interval are included in Table S1. Individual crossovers in five of the newly identified hotspots (shown in Table S1) were sequenced to determine exact locations of the chromatid exchange points within the limits of resolution provided by the locations of internal SNPs.

### Regional Variation of Recombination Activity along Chr 1 at 225 kb Resolution

In total, the sex-averaged genetic map length of Chr 1 in the B6xCAST cross was 90.9 cM, which represents an average rate of 0.469 cM/Mb across 193.8 Mb, excluding the centromere adjacent 3 Mb for which no sequence information is available according to NCBI sequence build 36.

**Figure 1:**
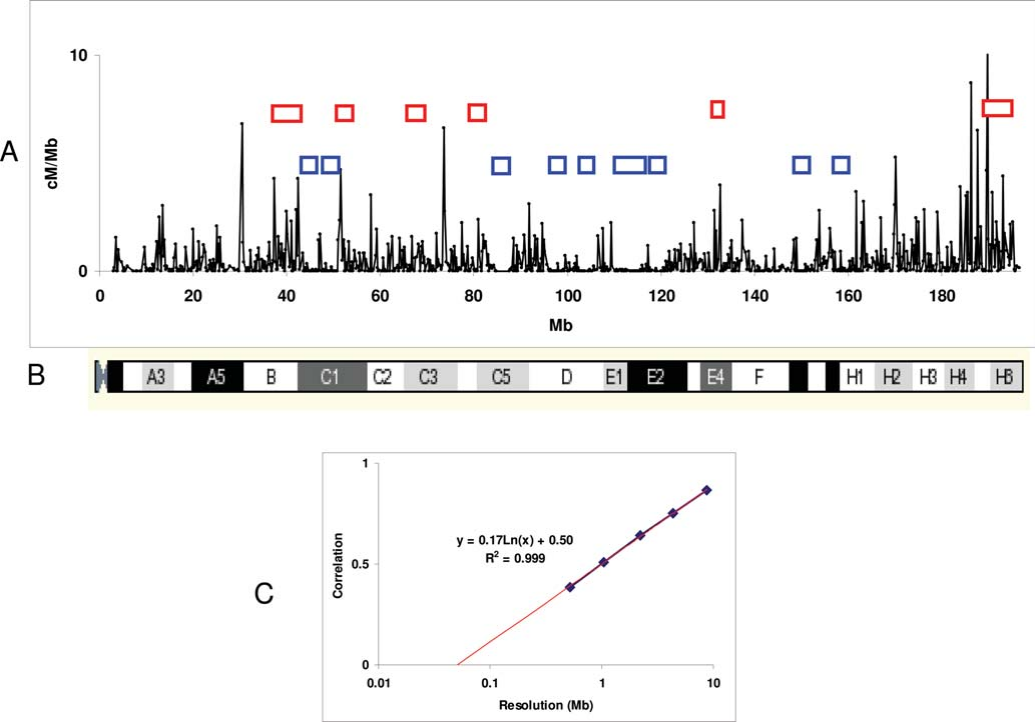
Recombination map of Chr 1. A. Sex-averaged recombination map of Chr 1 in C57BL/6J×CAST/EiJ cross. Boxes represent runs of consecutive intervals showing recombination (red) or no recombination (blue). B. Cytological map of Chr 1 (from ENSEMBL). C. Correlation between recombination rates in C57BL/6J×CAST/EiJ backcross and HS mice at different resolution. The red line represents the best fitting logarithmic trend extrapolated to zero correlation. The best fitting function and its correlation coefficient are shown, indicating that correlation between the two crosses approaches zero at distances around 0.05 Mb.

At 225 kb resolution, recombination activity was distributed very unevenly along the chromosome, forming alternating domains of higher and lower activity (Figure 1A). Recombination activity was found in only 64% of all intervals along the chromosome, the remaining 36% being completely devoid of recombination. In several places along the chromosome, recombination activity tended to be clustered in runs of consecutive intervals all of which were active, forming “torrid zones”. The most concentrated of them were 1.4–6.1 Mb long and were located at 37–41 Mb, 51–52.4 Mb, 72–74.8 Mb, 81.6–83 Mb, 131.4–132.8 Mb, and 189.5–195.6 Mb (red boxes in Figure 1A).

Correspondingly, intervals devoid of recombination activity tended to cluster in “cold zones”, the largest of which was over 6 Mb long. These were most prominent around 44.6–46.8 Mb, 48.6–51 Mb, 84.8–88.0 Mb, 96–97.8 Mb, 102.6–105.6 Mb, 110–116 Mb, 119–121.6 Mb, 149.2–151.4 Mb, 158.6–160.2 Mb (blue boxes in Figure 1A).

We did not detect any significant correlation along the chromosome between the locations of torrid and cold zones and traditional cytological banding patterns (Figure 1B).

### Conservation of Regional but not Local Variation in Recombination Rates

To test the extent to which the recombination properties of a chromosome are evolutionarily conserved, we compared our results, obtained in a cross of only two strains, with the recombination map of Shifman et. al. [17]. The Shifman map was prepared at an average 550 kb resolution using the progeny of heterogeneous stock (HS) mice which merge the genetic backgrounds of eight mouse strains, including C57BL/6J but not CAST/EiJ. The two crosses have similar regional distribution of recombination along the chromosome, but do not share a substantial fraction of hotspots, if any.

Regional conservation between the two crosses was indicated by the significant correlation of recombination rates along the chromosome when tested at long intervals (*r* = 0.87 at 8.75 Mb resolution, Pearson correlation). However, this correlation decreased markedly when smaller intervals (4.4 Mb, 2.2 Mb, 1.1 Mb and at the maximum resolution of 0.55 Mb) were compared (Figure 1C). At the half-megabase scale, we found only a weak regional correlation (*r* = 0.38).

These estimated correlations are somewhat attenuated by the sampling variation in the estimates of recombination rates, and this attenuation increases at higher resolution, since the sampling variation is greater at higher resolution (due to smaller numbers of observed recombination events in smaller intervals). But for the sample sizes in these studies, the attenuation in the estimated correlations is negligible (on the order of 1/1000), and so cannot account for the large observed decrease in correlation from the 8.75 Mb scale to the 0.55 Mb scale.

Long regions of very low or no recombination were evident in both crosses and provided the strongest parallels between the crosses. These regions include those around 43–50 Mb, 96–106 Mb, 111–116 Mb and several smaller regions between 141–152 Mb. The lack of recombination in these regions cannot be attributed to inversions, which would prevent the survival of recombinants. Two main reasons speak against this possibility. First, some parents in the mixed genetic background will inevitably have the same orientation of the region in question if it were inverted in some of the eight strains, and therefore recombination would be detected in their progeny. Second, some intervals in these regions are not totally devoid of recombination in both crosses but have very low rates.

### Effects of Genetic Background on Overall Recombination Rates

In addition to local variation in recombination rates, genetic background also plays a role in determining overall recombination rates. The genetic map length of Chr 1 was ∼31% higher in HS mice than in our two-strain cross. The reasons for this significant difference are uncertain. The lack of local correlation indicates that this difference is not simply due to an increased use of the same hotspots in HS mice. The present genetic data [22] agree with counts of the average number of chiasmata per meiosis during spermatogenesis among inbred strains [34] and counts of MLH1 foci marking sites of crossing over on Chr 1 [35]. It might be possible that recombination in a very heterogeneous genetic background is quite different from that seen in crosses of inbred strains. The importance of genetic background in recombination is also suggested by substantial differences between the crosses' recombination rates at specific intervals. For example, in the 24.7 Mb region that was mapped at considerably greater resolution (see below), recombinational activity was often present in one mouse cross (B6xCAST or HS) but not the other.

### Positioning Relative to Genes, Exons and Transcription Start Sites

We found an overall positive correlation between gene density and recombination along the entire chromosome over megabase distances (*r* = 0.557 at 10 Mb). However, this effect diminished over shorter distances (*r* = 0.164 at 500 kb) (Table 2). At 200 kb, the correlation was low (*r* = 0.079) but statistically significant. Moreover, this positive correlation was not uniform along the chromosome but was restricted to only some regions, and statistically significant only for the region between 100–150 Mb (maximum correlation *r*  = 0.877 at 5 Mb for the sex-average data). In this region, the positive correlation was still detected, and statistically significant, at 200 kb (*r* = 0.278). For the first and second 50-Mb segment (3–50 and 50–100 Mb), the correlation was positive but not statistically significant, whereas the correlation for the last region (150–194 Mb) was slightly negative up to 2Mb but not statistically significant. The 24.7-Mb part of the last segment was mapped to higher resolution (see below) and showed slightly negative correlation between gene density and recombination at 200 kb which disappeared at 50 kb.

**Table 2:** Correlation between gene density and recombination rates.

Sex	200	kb	500	kb	1	mb	2	mb	5	mb	10	mb
	Chr 1 entire											
	*r*	*p*	*r*	*p*	*r*	*p*	*r*	*p*	*r*	*p*	*r*	*p*
Female	**0.093**	**0.000**	**0.184**	**0.001**	**0.206**	**0.001**	**0.308**	**0.000**	**0.482**	**0.000**	**0.678**	**0.001**
Male	**0.055**	**0.045**	**0.126**	**0.011**	**0.141**	**0.023**	**0.255**	**0.005**	0.250	0.064	0.327	0.107
Sex-Average	**0.079**	**0.007**	**0.164**	**0.001**	**0.187**	**0.005**	**0.304**	**0.001**	**0.379**	**0.009**	**0.557**	**0.005**
	3–50 Mb											
Female	**0.131**	**0.030**	0.165	0.051	0.155	0.128	0.096	0.304	0.377	0.131		
Male	0.079	0.115	0.172	0.053	0.146	0.142	0.120	0.251	0.062	0.352		
Sex-Average	0.115	0.051	**0.180**	**0.036**	0.161	0.121	0.117	0.285	0.209	0.238		
	50–100 Mb											
Female	0.019	0.771	0.072	0.487	0.075	0.614	0.166	0.438	0.626	0.053		
Male	0.074	0.251	0.084	0.414	0.112	0.449	0.336	0.109	0.600	0.067		
Sex-Average	0.055	0.396	0.085	0.409	0.103	0.487	0.286	0.176	**0.674**	**0.032**		
	100–150 Mb											
Female	**0.295**	**0.000**	**0.405**	**0.000**	**0.495**	**0.001**	**0.696**	**0.000**	**0.876**	**0.000**		
Male	**0.191**	**0.007**	**0.299**	**0.006**	**0.419**	**0.003**	**0.558**	**0.003**	**0.821**	**0.003**		
Sex-Average	**0.278**	**0.000**	**0.403**	**0.000**	**0.505**	**0.000**	**0.689**	**0.000**	**0.877**	**0.001**		
	150–197 Mb											
Female	−0.035	0.317	0.051	0.321	−0.010	0.469	−0.019	0.499	0.576	0.068		
Male	−0.058	0.225	0.007	0.440	−0.096	0.305	−0.234	0.165	0.207	0.251		
Sex-Average	−0.053	0.218	0.025	0.403	−0.071	0.351	−0.175	0.248	0.433	0.138		
	168.8–193.5 Mb											
	50	kb	100	kb	200	kb						
Female	−0.007	0.427	−0.037	0.303	−0.067	0.238						
Male	0.018	0.335	0.010	0.430	−0.080	0.204						
Sex-Average	0.010	0.412	−0.008	0.460	−0.081	0.201						

*r* represents correlation coefficient, *p* is the probability calculated by bootstrapping. The correlations with *p*<0.05 are shown in **bold**.

Recombination tended to avoid gene deserts larger than 1.5 Mb but showed a tendency of clustering at their borders. The average rate in large gene deserts totaling 59.77 Mb (shown in Figure S1) was 0.26 cM/Mb compared to 0.55 cM/Mb in the remaining 134.02 Mb of non-deserts (*p*<10^−99^ by χ^2^ test) and 0.467 cM/Mb over the entire chromosome. The average rate was 0.80 cM/Mb in the 0.5–0.7 Mb border regions surrounding large gene deserts (*p*<10^−51^) and rapidly decreased beyond that to become statistically indistinguishable from the average chromosome rate (*p* = 0.596).

Similar correlation was found over the entire chromosome between exon density and recombination (*r* = 0.566 at 10 Mb and *r* = 0.126 at 500 kb, Table S2) and transcription start sites and recombination (*r* = 0.585 at 10 Mb and *r* = 0.121 at 500 kb, Table S3). However, the correlation was not statistically significant at 200 kb (*r* = 0.043, *p* = 0.101 for exons and *r* = 0.026, *p* = 0.204 for transcription start sites). In these two comparisons, most of the positive correlation was statistically significant for the region between 100–150 Mb but not for the rest of the chromosome. In the 24.7-Mb region mapped to higher resolution, both exon density and transcription start sites were slightly negatively correlated with recombination down to 50 kb (*r* =  −0.045 and *r* =  −0.071, respectively) and this effect was statistically significant for transcription start sites (*p* = 0.021).

Two striking examples of torrid zones that occur in large introns provide evidence that recombination is not restricted to intergenic regions. The first one consists of at least six hotspots in the 218-kb long second intron of *Pbx1* (pre B-cell leukemia transcription factor 1, located at 169.995–170.268 Mb, NCBI Build 36), which is also a hotspot for translocations associated with acute lymphoblastic leukemia in humans [36],[37]. The second torrid zone includes at least three hotspots in the 80-kb long third intron of *Esrrg* (Estrogen receptor-like receptor gamma, located at 189.309–189.915 Mb).

### Relative Abundance of Intervals with Differing Recombination Rates

**Figure 2:**
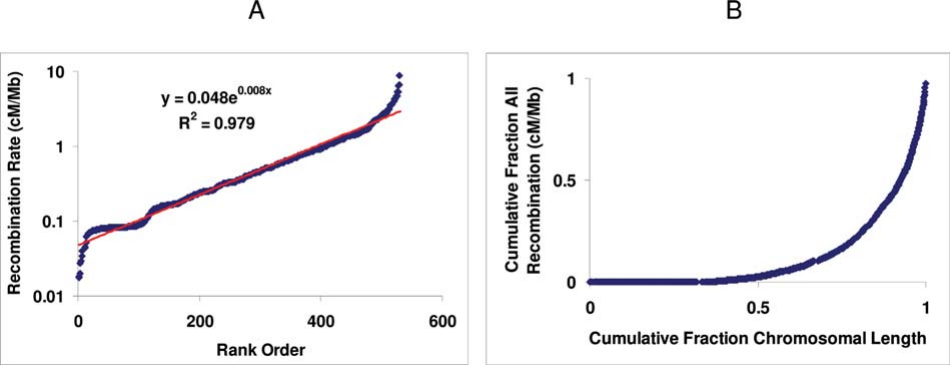
Distribution of recombination rates on Chr 1. A. Distribution of recombination in intervals of increasing rates (intervals lacking recombination are not included). The rates are presented in logarithmic scale to emphasize the exponential shape of the distribution. The deviation at the lower end of the distribution represents low-activity intervals mapped to a lower resolution. Red line represents the best fitting exponential function. The exponential function and its correlation coefficient are shown. B. Cumulative recombination as a function of chromosomal size. Both recombination rates and chromosomal length are expressed as fractions of the total. The intervals are in rank order of increasing recombination rate.

We observed a simple, negative exponential relationship between the crossover rate among intervals and the likelihood of seeing hotspots of that activity. Among intervals averaging 225 Kb in length, recombination rates (expressed as cM/Mb to correct for variations in interval length) varied continuously over almost three orders of magnitude, from 0.017 cM/Mb (the lower limit of detection in this cross) up to 10 cM/Mb. Intervals with differing recombination rate were not equally likely; instead, when they were placed in rank order of recombination activity, the rates were distributed in a simple exponential manner where *R_n_*, the recombination rate in the *n*th ranked interval was equal to *ke^cn^*, where *k* and *c* are constants (Figure 2A). Figure 2B, which is also an exponential function, describes the cumulative recombination rate among rank-ordered intervals. A similar exponential relationship for the cumulative recombination rate was reported by McVean et al [38] for the human genome.

These exponential relationships indicate that nearly 50% of all recombination activity occurred in only 7.6% of the intervals while 22.2% of the intervals accounted for 80% of all recombination activity. Similar findings that a high percentage of all recombination is concentrated in a small fraction of chromosome intervals have recently been reported for the human genome [39]. The interval fractions become even smaller with decrease in interval size (see below). This result, which suggests that the majority of all recombination events occur in a relatively small fraction of the chromosome, has important practical implications for genetic mapping strategies. The conclusion that follows is that a moderate size cross should be optimal for mapping genes and QTLs because adding more offspring will not substantially increase the resolution power. The result provides an experimental ground to something that mouse geneticists have known intuitively for some time-if a gene cannot be mapped with the first few hundred offspring, the best strategy is to move to another cross if that is at all possible.

### High Resolution Mapping in the Telomere-Proximal 24.7 Mb

**Figure 3:**
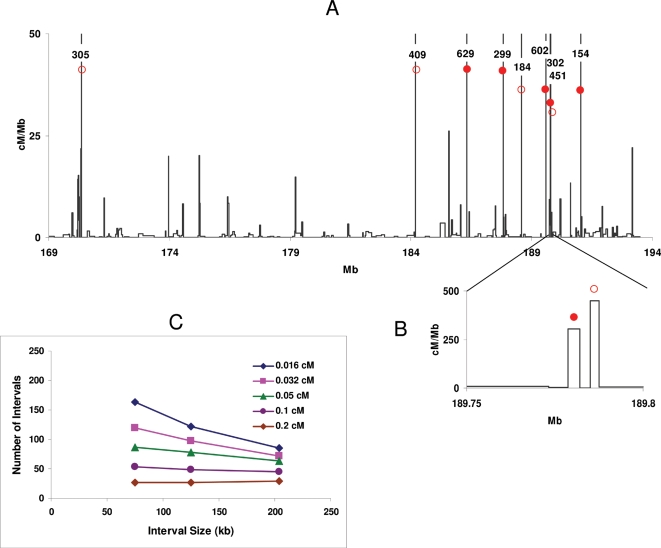
Fine mapping of recombination activities. A. Sex-averaged map of the region of 168.8–193.5 on Chr 1. Recombination rates in intervals that are off scale are shown as numbers over each interval. The red circles mark newly identified hotspots; full circles, hotspots that were sequenced through to determine the fine positioning of crossover exchanges. B. Hotspots in the third intron of *Esrrg* (189.75–189.8 Mb). C. Number of intervals containing recombination activity higher than given thresholds at different interval size. The threshold levels are shown in the legend.

High-resolution mapping further emphasizes the uneven distribution of recombination activities among intervals (Figure 3A and Figure S2).

The 24.7-Mb telomere-proximal segment between 168.8–193.5 Mb had a genetic length of 22.7 cM. This accounts for a relative recombination rate of 0.92 cM/Mb, which is about twice the average rate of the entire chromosome. When it was mapped further to an average resolution of 75 kb, the distribution of recombination activities among intervals remained continuously variable as in the 225 kb intervals. However, as expected from the punctate location of hotspots, a smaller fraction of the genome-52% compared to 64% at 225 kb resolution–contained all recombination. Indeed, 50 percent of all recombination occurred in 16 intervals spanning only 1.8% of the segment length, with each of these intervals having an activity of 0.34 cM or more.

Recombinations in eight of these sixteen most active intervals were mapped down to 20–45 kb resolution while those in the remaining eight intervals marked with red circles on Figure 3A were mapped down to ∼3 kb resolution. All but one of the eight intervals contained a single hotspot, which was separated from the closest adjacent hotspot by at least 30 kb of sequence. The notable exception was the presence of two hotspots only 5 kb apart in the third intron of the *Esrrg* gene (Figure 3B).

Distances between adjacent intervals with recombination rates of 0.34 cM or more varied over three orders of magnitude in genomic terms, ranging from 5 kb to 5 Mb (1.52 Mb on average). The variation was much smaller in genetic terms, from 0.37 to 2.44 cM, or an average of 1.26 cM.

### Total Number of Hotspots in the Mouse Genome

As interval sizes become smaller, it becomes increasingly likely that an interval contains only one hotspot. This provides a means of estimating the total number of hotspots in this 24.7-Mb segment, and by extension the total number in the genome. For this, the number of intervals showing any recombination activity was plotted as a function of interval size and the resulting trend lines extrapolated to a 5kb interval size, the minimal distance we found between adjacent individual hotspots (Figure 3C, results summarized in Table S4). This yielded an estimate of one hotspot per 108 kb on average, or about 228 hotspots accounting for all recombination in this segment among 6028 meioses. As expected from the exponential relationship described above, more active hotspots occur less frequently. On average, those with rates higher than 0.1 cM are likely to occur once per 425 kb, and those with rates higher than 0.2 cM, about once per megabase. These results are obviously tempered by the fact that they were obtained for one genetic combination in a region of the genome whose recombination rate is higher than the genome wide average.

To the extent this region is representative of the rest of the genome, its hotspot density provides an estimate of the total number of hotspots in the entire mouse genome that are active in this B6xCAST cross. We have made this estimation by relating the genetic length of the 24.7-Mb region to the total genetic length of the mouse genome. We assume that genetic lengths (measured in cM) will be more relevant than physical lengths (measured in Mb) because of the uneven distribution of recombination along the chromosome and the existence of long regions devoid of recombination. This calculation, using the Dietrich et al [30] sex-average map length of 1361 cM for the same C57BL/6JxCAST/EiJ cross, results in an estimate of about 13,670 hotspots (228/22.7×1361) across the mouse genome.

A recent study [40] typing 8.23 million SNP markers detected about 40,000 haplotype blocks in 12 classical inbred mouse strains based on ancestry inferred from representative strains of the four main mouse subspecies. Although the haplotype block boundaries were not always well defined, to the extent that they represent bona fide historical sites of recombination, the scales of these two estimates are not far apart. Our study should be considered a minimum estimate as it measured recombination from contemporary hotspots in one generation of a cross involving only two inbred strains, and was limited by the sensitivity of detection of 6028 meioses. The estimate of Frazer et al [40] suggested a higher number of hotspots in the genome of classical mouse inbred strains because it is not limited to contemporary hotspots and reflects the behavior of historical hotspots generating recombination over many generations in a variety of genetic backgrounds.

The most recent estimate [41] using more than 3.1 million SNPs has identified 32,996 hotspots in the human population, which is in the range of these estimates for the mouse genome.

### Sex Specificity of Recombination

The two sexes differed at all levels of organization of recombination. Overall recombination rates were higher in females than males; recombination was distributed differently along the chromosome in males and females, and there were also sex-specific hotspots.

**Figure 4:**
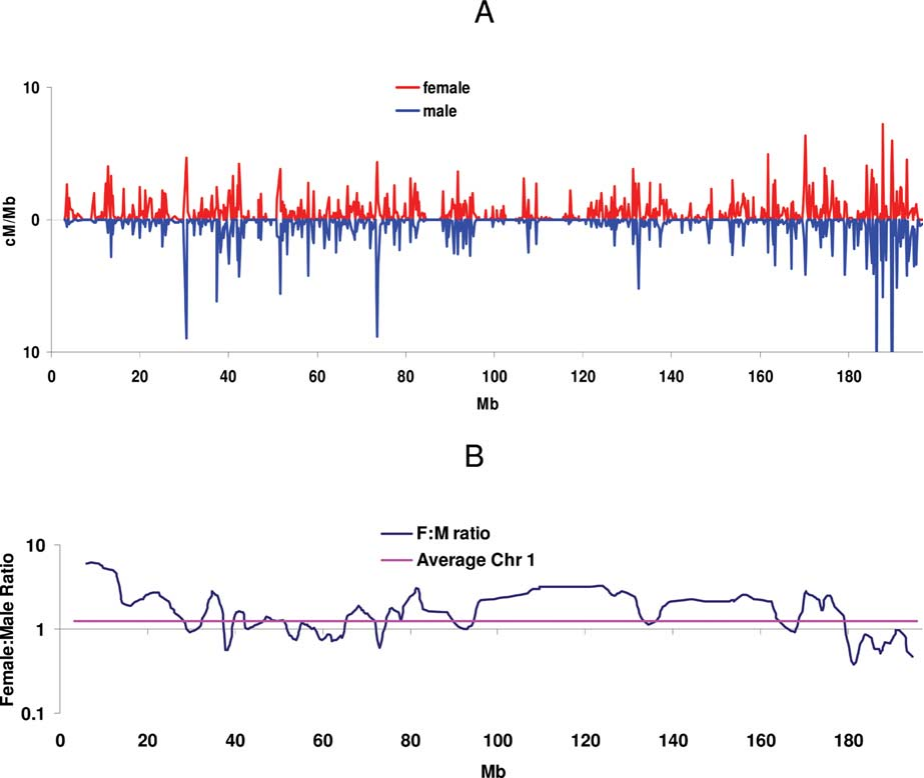
Sex specificity of recombination. A. Sex-specific recombination map of Chr 1. Red line, female recombination rates; blue line, male recombination rates. B. Female:male ratio along the chromosome. Dark blue line: female:male ratio; purple line: sex-averaged recombination rate over the entire Chr 1.

The female recombination map of Chr 1 was 99.5 cM, or 1.21 times longer than the male map which was 82.3 cM, with average recombination rates over the entire chromosome of 0.51 and 0.42 cM/Mb, respectively. These differences were statistically significant (*p*<10^−6^ by Fisher's exact test). Among 225 Kb intervals, there was an overall positive correlation between female and male rates (*r* = 0.64) along the chromosome. This correlation did not change significantly at larger interval sizes up to 8 Mb. The underlying reason why the correlation did not increase with interval size was the substantial variation in distribution of recombination along the chromosome (Figure 4A), which included differences in both the number and relative recombination activity of intervals.

Recombination activity was spread over a larger fraction of the chromosome in females than in males. In females, 57.1% of intervals were recombinationally active compared to only 42.2% in males (a ratio of 1.35). This differential was apparent at all activity levels; 80% of all activity occurred in 23.2% of female versus 13.6% of male intervals, and 50% occurred in 8.23% of female versus 4.65% of male intervals.

These sex differences in the relative rates of recombination were regionally controlled (Figure 4B). Female recombination rates were higher in the centromere-proximal 27 Mb and in the region between 79–178 Mb, whereas male recombination rates were higher in the telomere-proximal 178–197 Mb region and generally, but not in the entirety, of the region between 27–79 Mb.

**Figure 5:**
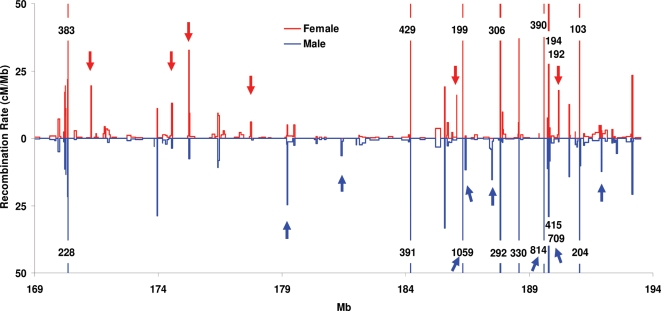
High-resolution sex-specific recombination maps of the interval between 168.8–193.5 Mb. Recombination rates in intervals that are off scale are shown as numbers over each interval. Red arrows: hotspots predominantly active in females; blue arrows: hotspots predominantly active in males.

To study regional effects in more detail, we examined the switch between higher female and higher male recombination found in the fine-mapped 24.7 Mb sub-telomeric region. Female recombination rates were generally higher than those in males in the region between 169–178 Mb, with an abrupt transition to the opposite case in the adjacent region between 178–194 Mb where males had higher recombination (Figure 5 and Figure S3). Interestingly, the switch occurs in a region of very low recombination in both sexes. Overall, the difference between the two sexes was highly significant over the entire region (*p*<10^−4^).

Although the sexes share a substantial fraction of hotspots, there are many considerable differences in activity. Commonality of hotspot usage was indicated by the observation that comparisons at multiple interval sizes did not change the correlation between the two sexes (*r* = 0.62). However, there were also specific sex differences in hotspot activity that were independent of regional control. Among the 28 intervals with sufficiently high recombination (>0.2cM) to provide sufficient numbers of crossovers for statistically significant analysis, 18 showed sex-specific differences after adjustment for multiple testing (Table 3). Among these 18, eleven showed at least some activity in both sexes, seven being markedly more active in females and four in males (*p*<0.01, *q*<0.1). Seven of the 18 were detected in only one sex, four in females and three in males. The latter group indicates that some hotspots may be truly sex specific, or at least that the differences in their activity are so great (>10 times) that recombination was not detected in the low-activity sex even in several thousand meioses.

**Table 3:** Sex-specificity of hotspots in the 168.8-193.5 Mb region.

	Number of Recombinants		Significance	
Hotspot Location (Mb)	female	male	*p* *	*q* **
171.3	15	0	0.000	0.000
186.3	17	88	0.000	0.000
189.8	19	63	0.000	0.000
187.4	0	15	0.000	0.007
190.2	13	1	0.000	0.007
174.4	20	5	0.001	0.016
176.5	9	0	0.001	0.016
181.3	0	11	0.001	0.016
175.2	17	4	0.002	0.023
186.4	1	13	0.003	0.031
179.2	4	20	0.003	0.031
177.7	10	1	0.003	0.031
171.7	7	0	0.006	0.049
171.9	14	3	0.007	0.056
191.0	0	8	0.008	0.063
176.7	6	0	0.010	0.073
170.3	40	24	0.012	0.085
170.6	8	1	0.015	0.099

*: *p* values are calculated by Fisher's exact test.

**: *q* values are calculated as described in [56].

Importantly, this sex specificity of individual hotspots is not constrained by regional controls. For example, the hotspot at 173.967 Mb is more active in males despite lying in the midst of a female predominant region, and the hotspot at 190.204 Mb, which is considerably more active in females, nevertheless lies in a male predominant region.

To address the broader question of how the total numbers and relative activity of hotspots differ between male and female meioses, we compared the two sexes across the female and male predominant segments of the subtelomeric 24.7 Mb region by extrapolating the resolution dependent trend lines for activity down to 5 Kb. Interestingly, the two regions gave distinct answers; greater female recombination in the proximal segment largely resulted from an increased number of hotspots, whereas in the distal segment, greater male recombination was primarily the result of increased recombination in a comparable number of hotspots (Table 4). In the proximal 9.8 Mb, where females had twice the recombination rate of males (9.0 cM vs. 4.2 cM), they had twice as many hotspots as well (72 vs. 34) that were somewhat more active, while in the distal 16 Mb where females have a significantly lower recombination rate than males (12.4 cM vs 19.8 cM), there were similar numbers of inferred hotspots (91 vs. 88) in the two sexes, but males had higher average recombination rates per hotspot.

**Table 4:** Inferred number of hotspots in females and males in the interval of 168.8–193.5 Mb.

			Females			Males	
Genomic Region (Mb)	Hotspot Activity (cM)	Number of Hotspots		Density (HS/Mb)	Number of Hotspots		Density (HS/Mb)
168.8-193.5	>.032	163		6.6	122		4.9
	>.05	105		4.3	82		3.3
	>0.1	81		3.3	54		2.2
	>0.2	32		1.3	30		1.2
	**Rec. Rate (cM)**		21.5			24.0	
168.8-178	>.032	72		7.3	34		3.5
	>.05	48		4.9	23		2.3
	>0.1	28		2.9	16		1.6
	>0.2	13		1.3	4		0.4
	**Rec. Rate (cM)**		9.0			4.2	
178-193.5	>.032	91		5.9	88		5.7
	>.05	57		3.7	59		3.8
	>0.1	33		2.1	38		2.5
	>0.2	19		1.2	26		1.7
	**Rec. Rate (cM)**		12.4			19.8	

These sex differences largely apply to lower activity hotspots, those less than 0.2 cM. The inferred numbers of hotspots with rates of up to 0.2 cM were significantly higher in females than in males over the entire 24.7 Mb (Table 4). However, this inequality did not hold for higher activity hotspots; both sexes had the same number of hotspots more active than 0.2 cM.

### Distinct Chromatid Control at Individual Hotspots

Fine mapping of crossover exchange points within hotspots made it possible to identify the parental chromosome initiating recombination and thereby show that the two parental chromatids are under independent recombinational control.

**Figure 6:**
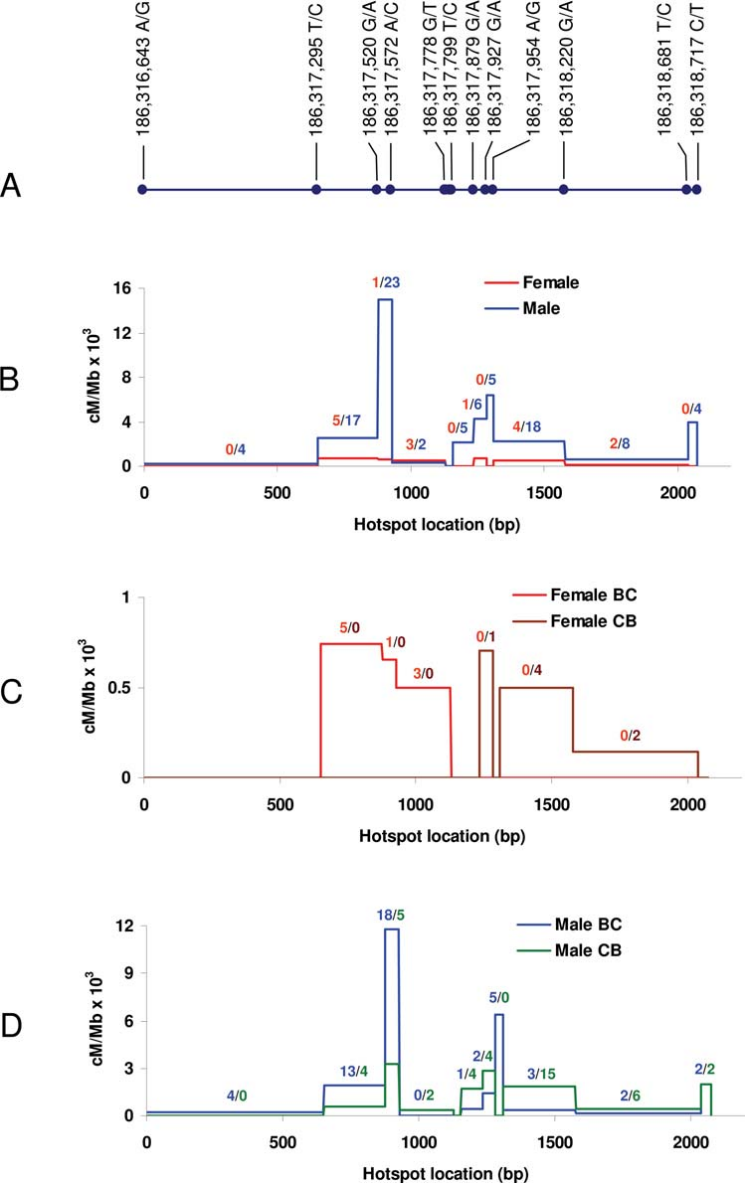
Distribution of crossover exchange points at hotspot 186.3. A. Physical positions of the SNPs used to determine the crossover exchange points according to NCBI Build 36. In panels B, C and D, the left end (0) corresponds to 186,316,643 A/G. B. Distribution of crossover exchange points in female and male progeny. The number of crossovers in each interval is shown. Red, females; blue, males. C. Distribution of reciprocal crossovers (B-C and C-B) in female progeny. The number of crossovers in each interval is shown. Red, B-C; tan, C-B. D. Distribution of reciprocal crossovers (B-C and C-B) in male progeny. The number of crossovers in each interval is shown. Blue, B-C; green, C-B.

The locations of all 457 crossover events in five of the nine hotspots mapped to <3 kb resolution (marked with full red circles on Figure 3A) were further mapped using all available SNPs. In each case, the sites of crossing over were distributed over distances ranging from 500 to 2000 bp, which is a typical size for a hotspot [3] (Dataset S1). In some cases, recombination activities were distributed along the entirety of the hotspots regions following a single normal distribution, but in others they appeared to be the sum of two overlapping bimodal distributions. Distinguishing between the two distributions depended on the availability of SNPs for precisely mapping recombination events near the hotspot center. When such conveniently positioned SNPs were available, we observed that crossover events were predominantly located at the two sides of the hotspot, with very little or no recombination at the center (Figure 6B). According to the currently valid models of recombination, bimodal distribution will be observed when double strand breaks initiate in very narrow regions, and the crossover exchange points which are located at the sites of resolution of the Holliday junctions migrate sufficiently away from the initial sites of double strand breaks. Our finding that a bimodal distribution was observed when the necessary SNPs were available for detection suggests that this is likely to be the case for most hotspots.

For the hotspot at 186.3 Mb, the availability of particularly suitable SNPs (Figure 6A) allowed us to deduce that for this hotspot the B6 and CAST chromatids are under independent, sex-specific recombinational control. The sites of crossing over within the hotspot were quite different when the crossover products were B proximal-C distal v. C proximal-B distal. This was true for F1 animals derived from both reciprocal crosses, i.e. there were no imprinting effects. Among the 16 crossovers arising in female meioses, all B-C exchange points were positioned centromere-proximal to the center of the hotspot, whereas all C-B recombinants crossed over in the centromere-distal part. Thus, the center of the hotspot was of CAST origin in all crossovers (Figure 6C), indicating that, in this cross, recombination events in females only initiated on the B6 chromosome [5]. In males, which have 5.6 times higher recombination at this hotspot, there was also a strong bias towards initiation on the B6 chromosome, although the effect was not absolute. Crossover events of both types were distributed on both sides of the central region, indicating that recombination could initiate on either parental chromatid (Figure 6D). However, initiation on the B6 chromatid was 2.5 times more frequent than on the CAST chromatid.

Our results for the 186.3 hotspot clearly show that the overall control of recombination at a hotspot is the sum of distinct controls for each chromatid, and that this distinction applies to issues of both sex specificity and absolute recombination rates.

### Imprinting of Recombination Activities

Examining 225 Kb intervals over the entire chromosome to compare F1 hybrids derived from the reciprocal crosses of B6xCAST and CASTxB6 provided statistically significant evidence for parent-of-origin effects on recombination activities in both sexes (*p* = 0.013 for reciprocal males and *p* = 0.009 for reciprocal females). The direction of imprinting was not uniform, and imprinting was only detected by finding a statistically significant excess of hotspots showing a preference for recombination in one direction of the cross or the other. In no case did we find absolute imprinting, where recombinants were significantly absent from one direction of the cross. A statistically significant difference was also detected in the fine mapped 24.7-Mb region of the chromosome in males (*p* = 0.001), but the difference was only marginally significant in females (*p* = 0.07). None of the higher activity hotspots in this region showed significant parent-of-origin effects after correction for multiple testing; rather, imprinting effects were restricted to medium- and low-activity hotspots. (See Tables S5 and S6).

However, although we detected slight but significant cumulative differences between reciprocal crosses in 225 Kb intervals in both female and male meiosis, and in male meiosis in the telomere-proximal 24.7 Mb, no one interval gave significant evidence for a difference in recombination rate between the reciprocal crosses. It is likely that the effects may be subtle and only recognizable statistically when data is accumulated across large chromosomal regions. Individual intervals, when considered on their own, showed recombination rate differences between the reciprocal crosses that could reasonably be explained by chance variation, but overall there were many more intervals with suggestions of recombination rate differences than could reasonably be explained by chance variation.

### Gene Conversions and Genetic Interference

Additional data obtained from the backcross animals provided the first genetic evidence in mammals that genetic interference, which regulates the spacing of crossovers, does not affect the relative locations, one to the other, of the two distinct outcomes of the recombination process, crossing over and gene conversions not associated with crossing over.

Gene conversions arising in male meioses were detected in three of the fine-mapped hotspots by genotyping every SNP across each hotspot among 1365 male backcross progeny (Table 5). Only eleven conversions were found, six conversions not associated with crossovers (noncrossovers) and five conversions associated with simultaneous crossovers at the same hotspot. In the best mapped hotspot at 186.3 Mb, all five events we detected were positioned in the central part of the hotspot. The three noncrossovers were located between positions 1135–1311 bp on Figure 6B, and the two conversions associated with crossovers spanned between positions 877–1311 bp. For all three hotspots, the apparent frequencies of non-crossover conversions were lower (5–11 times) than crossover frequencies at the same hotspots, however these ratios must be interpreted with caution as while we were able to detect all crossovers, we were only able to detect the sample of conversions occurring at sites of available SNPs. The relative ratios of crossovers to noncrossover conversions in several human and mouse hotspots have shown considerable variation, from more than 12∶1 to 1∶4 [2],[5],[25],[42]. Given the positions of the available markers, the actual conversion frequencies could be much higher than detected. From SNP locations we could deduce that the minimum-maximum length for noncrossover conversion tracts was 9–279 bp. In contrast, conversion tracts associated with crossing over at the same hotspots had a minimum-maximum span of 199–1196 bp. Both estimates are of similar scale to those reported at the human DNA3 hotspot, 55–290 bp for conversion tracts not associated with crossovers and ∼460 bp for conversion tracts associated with crossing over [25].

**Table 5:** Crossover and non-crossover rates at three hotspots in 1365 progeny of a male backcross.

Hotspot	186.3	187.8	189.78
NCR conversions	3	1	2
CR conversions	2	1	2
Crossovers	31	11	10
NCR Rate	0.002	0.001	0.001
CR Rate	0.023	0.008	0.007
NCR/CR Ratio	0.097	0.091	0.200

The six progeny chromosomes carrying noncrossover conversions contained seven crossovers located elsewhere along the chromosomes. In four cases the distances between crossovers and conversions were significantly longer, 95–120 Mb, than the minimal male interference distance of 57 Mb between two crossovers observed in the 3026 male meioses used in this study [22]. However, in three cases the crossovers and conversions were only a few megabases apart, the closest distance being 1.12 Mb. We conclude that the process of genetic interference limiting the proximity of crossovers, one to another, does not limit the proximity of crossovers and non-crossover conversions. Our finding is in agreement with the lack of interference between crossovers and non-crossover conversions originally found in yeast [43].

## Discussion

This study presents the first high-resolution, comprehensive investigation of recombination as it occurs over an entire mammalian chromosome in a defined genetic background. As such, it provides material for further research, and as one might hope, generates as many questions as it provides insights.

The distribution of recombination along chromosome 1 provides genetic evidence that at least two levels of control regulate positioning of crossover events in mice; one is at a regional scale and another at the level of hotspot activity. This result is most apparent when comparing the genetic map created in the cross between B6 and CAST with the map reported for HS mice [17]; the two crosses share regional patterns of recombination but few if any hotspots. McVean et al [38], using linkage disequilibrium data, previously came to the same conclusion regarding human recombination.

In the case of mice, this substantial regional variation in the distribution of recombinational activities allowed us to examine the sex specificity of this phenomenon. Male recombination is concentrated at the telomere-proximal region, whereas female recombination is more evenly distributed along the chromosome. Importantly, however, the two sexes appear to share similar pattern of megabase-scale regions containing or lacking recombination as well as substantial portion of their hotspots within this regional variation, although at different activity levels.

The question then arises as to what the source of this regional variation might be as it is only to some extent related to exon density and not related to the other obvious biological feature of chromosomes-cytological banding patterns. The existence of alternating regions of high and low recombination suggests that regional recombinational activity might be an intrinsic property of genomic content. However, the general observation of high male recombination in subtelomeric regions suggests that positional effects, i.e. regional location relative to centromere and/or telomere may also play a critical role. Deciding between these possibilities may require comparisons of recombination patterns among chromosomes and between organisms carrying substantial chromosomal rearrangements.

Our data clearly show a multi-layered control of sex differences in recombination. First, averaging across the entire genome, females have an overall higher recombination rate than males. We have shown in another study [22] that the underlying reason for this is the crossover interference distance, which is shorter in females than in males when measured in megabases, allowing female chromosomes to accommodate more multiple crossovers. This difference in interference distances corresponds to differences in the length of the synaptonemal complex at pachynema [35],[44] and the synaptonemal complex length covaries with crossover/chiasma numbers [45]. Interference distances are the same in the two sexes when measured in microns of synaptonemal complex length, but the lesser compaction of female chromosomes results in fewer Mb of DNA per micron of length and hence greater opportunities for multiple crossing over.

The sexes also differ in the regional control of crossing over and the positioning of crossovers along the chromosome. Female recombination is distributed more evenly along the chromosome with alternating regional domains of higher and lower activity from centromere to telomere. In contrast, male recombination is more strongly localized, with two prominent peaks–one at the telomere-proximal region between 178–197 Mb and another at 27–79 Mb. It should be noted that the distance between the centers of the two male peaks equals the average intercrossover distance in male meiosis [22].

The sexes also differ at the local level in the usage of hotspots. Increased male recombination is associated with increased hotspots activity rather than an increase in the number of hotspots, whereas increased female recombination is associated with an increase in the number of hotspots of medium and low activity. These differences in hotspot usage are then reflected in the fact that the fraction of the chromosome (i.e. the number of 225 Kb intervals) exhibiting recombination is appreciably greater in females.

Finally, beyond these broad scale and regional effects there are truly sex-specific hotspots that may be found anywhere, including male specific hotspots in regions of predominantly female recombination and vice versa.

Our results examining mouse recombination show striking similarity to the features of sex specificity of recombination described in a human population-dramatic megabase-scale sex differences, similar overall use of hotspots by the two sexes, and examples of hotspots used mainly by one or the other sex [39].

The molecular origins of the sex effects must be complex, at the least involving differences in the nature of chromatin compaction during meiosis, the regional organization of chromatin, and sex-specific factors influencing the choice and activity of hotspots during meiosis. Given that the same chromosomal DNA sequences are the substrates for recombination in male and female meioses, these differences must reflect the existence of differentially transcribed, trans-acting factors controlling various aspects of recombination, but their identity is entirely unknown. Equally enigmatic are the biological functions and/or evolutionary selective pressures that underlie these differences. Do they have a primary function, or are they secondary consequences of other, underlying aspects of meiosis?

The exponential relationship between the frequency and activity of hotspots of different activity classes implies a probabilistic component to the determination of hotspot activity. This could result from a simple mechanism involving the accumulation of “units” that each contribute to the free energy requirement of hotspot activation. In the hope of promoting further discussion of what this “unit” might be, we here propose one possible formulation of the problem which suggests that an exponential function will be observed if two conditions prevail. The first condition requires that the relative activity of hotspots depends on the number of “units” they acquire. In this case, the probability of acquiring *u* units will be *P_u_ =  (P_1_)^u^*, where each unit has a nearly equal but independent probability of being acquired. The second condition would require that each unit contributes a nearly equal increment of free energy, so that the free energy available to initiate recombination, *ΔG,* is proportional to *u*. Then, given the familiar relationship *ΔG = −RT* ln*k*, *k* (which we interpret as proportional to the forward rate constant of the initiating step) becomes proportional to *e^ΔG^ = e^cu^*. This formulation has the utility of focusing attention on the challenge of identifying the physical nature of a “unit”, which in principle could represent anything from formation of a single hydrogen bond to the assembly and/or disassembly of nucleosomes.

We found fairly strong evidence that parent of origin effects, i.e. imprinting, influence hotspot behavior. However, this is not expressed in a simple on-off manner as it is in many cases of imprinting control of gene expression where one parental allele is virtually silenced relative to the other. The failure to detect any overall preference for one parental direction vs. the other (B6xCAST vs. CASTxB6) likely reflects variation among hotspots as to which parental chromatid initiates recombination more frequently and hence which parental direction is favored. The imprinting effect on recombination was only apparent as a tendency when combining data from across the chromosome and could not be detected at statistically significant levels at any single hotspot, even when taking the issue of chromatid specificity into account. In females, the hotspot at 186.3, which only activated on the B6 chromatid, failed to show any recombination bias between reciprocally generated F1 animals. Our finding is somewhat surprising because it has been well established that methylation imprints at maternally or paternally expressed genes are erased during primordial germ cell development [46],[47] and reestablished during gametogenesis. The possible role of imprinting in recombination has been discussed previously [48],[49]. Despite a lack of prior evidence that it does occur, these authors argued that imprinting should play a role in recombination as it is the only process in ontogenesis that requires recognition and contact between homologous chromosomes. Additionally, the possibility holds attraction as a means of enabling the distinction between sister and non-sister chromatids, an essential feature of meiosis.

Finally, we are left with one of the ultimate questions in recombination biology; what makes a hotspot a hotspot? Several aspects of this question have been elucidated in yeast [50] where three classes of hotspots can be distinguished. Unfortunately, although the identification of a series of new hotspots does provide new experimental material, we are still far from adequately answering this most critical question in mammals, which has already been addressed extensively with limited success by others [26],[51],[52]. The most definitive progress has been made in identifying nucleotide motifs that could explain a fraction of recombination activity based on LD data [52] and recently confirmed by crossover mapping [39].

Previously, elucidation of the possibilities for *cis* and *trans* regulation of recombination activity in mammals [3],[5],[23] has relied on qualitative data. In humans, much higher recombination in females than in males has been reported for the *TAP2* hotspot [53]. The most detailed investigation of *cis* and *trans* control of hotspot activity has involved the mouse *Psmb9* hotspot. Shiroishi et al [23] established that this hotspot is active only in female meiosis and only when in the context of a particular surrounding chromosomal segment. When the centromere-proximal part of the active segment was replaced, hotspot activity was lost. In males, replacing the centromere-distal segment resulted in additional hotspot activation. Baudat and de Massy[5] have extended this analysis to present evidence that *trans* as well as *cis* acting factors regulate *Psmb9* activity. The one refinement we can offer is the realization that, as exemplified by the hotspot at 186.3 Mb, the control of crossing over is chromatid specific. The control of a “hotspot” is, in effect, the sum of controls of the individual chromatids present at meiosis. Exploring this question in detail requires the ability to distinguish, quantitatively, the activity of each separate chromatid.

In conclusion, our data present a picture of recombination patterns along a chromosome that are controlled by a dynamic, complex regulatory system, with multiple levels of regulation depending on species identity, genetic variation, sex-specific mechanisms of recognition, and usage of specific hotspots. Only a fraction of all potentially available sites are used in a given F1 hybrid between two inbred strains, presumably as a function of the combined genetic contributions of both parents.

Improving our understanding of the structures and mechanisms bringing about these multiple layers of regulation for one of the most fundamental of biological processes is likely to cast light on several aspects of population genetics and evolutionary biology, as well as enhance our practical ability to define the genetic components of human disease.

## Material and Methods

### Strains, Crosses, and Genotyping

C57BL/6J and CAST/EiJ were obtained from The Jackson Laboratory, Bar Harbor, USA. F1 hybrids were produced by reciprocal crosses in which either strain was the female or male parent. These hybrids were then backcrossed to C57BL/6J and recombination was detected in their progeny. All parents and F1 hybrids were genotyped for three markers on each chromosome to ensure strain identity using DNA isolated from tail tips.

To prepare DNA for genotyping, mouse spleens were digested in 900 µl buffer containing 50 mM KCl, 10 mM Tris-HCl, pH 8.3, 2.5 mM MgCl_2,_ 0.1 mg/ml gelatin, 0.45% v/v Nonidet P40, 0.45% v/v Tween 20, and 60 µg/ml proteinase K overnight with occasional shaking. After digestion, the pH of the samples was adjusted by adding 100 µl of 100 mM Tris-HCl, pH 8.0. These digests were stored at −80°C. Samples were diluted 20x in 10 mM Tris-HCl, pH 8.0 for genotyping. All progeny were genotyped at 10 Mb resolution using previously described assays [54] for single nucleotide polymorphisms (SNPs) based on Amplifluor technology [55]. Individuals with a gap of >20 cM or >35 Mb between typed markers were omitted from subsequent analyses. Recombination was detected as a transition from homozygous to heterozygous genotype or vice versa. New Amplifluor assays were developed for the subsequent rounds of genotyping using the publicly available SNP database of the Mouse Phenome Project (http://phenome.jax.org/pub-cgi/phenome/mpdcgi?rtn=snps/door). In the second round, all recombinants detected were mapped at 200 kb resolution. In the subsequent rounds, recombinants were mapped to increased resolution until reaching the maximum hotspot resolution. In each round, the flanking markers from the previous round were retyped to confirm the validity of the recombinants. All detected conversions were confirmed by sequencing. This approach ensured extremely low error rate. A list of all markers used in this study is available as part of the Online Supporting Material (Table S1). The positions of all markers are in accordance with NCBI Build 36.

### Statistical Analysis

All the analyses were performed using R (http://www.r-project.org/) on the untransformed data (i.e. numbers of crossovers per interval). To compare recombination rates between groups (between the sexes, or between the two reciprocal crosses within one sex), first we tested whether there exists any difference in any interval across all intervals of the entire chromosome. An omnibus likelihood ratio test was used to compare the probability of the data if the recombination rate is allowed to be different across groups within each interval, versus the probability when the rates are forced to be the same across the groups for all intervals. A significant difference between the two groups indicates a difference in the recombination rate for at least one interval. The distribution and significance of the test statistics were determined via permutation method (>10,000 permutations). Then we tested the differences within individual intervals to see where the signal, if any, was coming from. Both likelihood ratio tests and Fisher exact tests were implemented and they produced similar *p*-values. These *p*-values were then transformed into *q*-values based on Storey and Tibshirani [56]. A *q*-value cutoff of 0.1 (equivalent to a false discovery rate (FDR) of 10%) was used to determine significant intervals.

### Correlation between Gene Density, Exon Density, Transcription Start Sites, and Recombination

The exon and transcript data was downloaded from the UCSC MySQL server (http://genome.ucsc.edu/FAQ/FAQdownloads#download29) using data from NCBI Build 36 of the mouse genome. The density is the fraction of the genome within transcribed sequences or exon coding regions, respectively, calculated in 50 Kbp blocks. Transcription start site density represented the number of 5′-gene ends per 50 kb. For the exon and transcript coverage, overlapping was treated as a continuous exon or transcript. Transcriptional starts only considered unique start sites; i.e., if two or more transcripts had a common start site, the site was only counted once. Correlation was calculated using the Pearson's product-moment correlation between the normalized recombination rate (cM/Mb) and the genomic feature (i.e., gene density, exon density, transcription start sites). The significance of the correlation was determined by 1000 bootstrap iterations, counting the number of correlations with an absolute value greater than the absolute value of the original correlation. Repetition of the bootstrap analysis found the results to be robust and no significant improvement was observed when using more than 1000 iterations.

## Supporting Information

Figure S1 Graphical representation of recombination, gene deserts, and exon density on Chr 1.
(0.07 MB PDF)

Figure S2 Fine mapping of recombination activities in the region of 168.8-193.5 on Chr 1 – number of recombinants.
(0.02 MB PDF)

Figure S3 Fine mapping of sex-specific recombination activities in the region of 168.8-193.5 on Chr 1 – number of recombinants.
(0.01 MB PDF)

Table S1 Number of recombinants detected in each tested interval in the four backcrosses.
(0.24 MB XLS)

Table S2 Correlation between exon density and recombination rates.
(0.17 MB DOC)

Table S3 Correlation between transcription start site density and recombination rates.
(0.17 MB DOC)

Table S4 Approximation of the number of hotspots with given activity.
(0.03 MB DOC)

Table S5 Direction specificity (imprinting) at hotspots in reciprocal female crosses.
(0.06 MB DOC)

Table S6 Direction specificity (imprinting) at hotspots in reciprocal male crosses.
(0.06 MB DOC)

Dataset S1 Sequences of the newly identified hotspots with SNPs between C57BL/6J and CAST/EiJ.
(0.07 MB DOC)

## References

[1] de Massy B (2003). Distribution of meiotic recombination sites.. Trends Genet.

[2] Baudat F, de Massy B (2007). Regulating double-stranded DNA break repair towards crossover or non-crossover during mammalian meiosis.. Chromosome Res.

[3] Jeffreys AJ, Kauppi L, Neumann R (2001). Intensely punctate meiotic recombination in the class II region of the major histocompatibility complex.. Nat Genet.

[4] Tiemann-Boege I, Calabrese P, Cochran DM, Sokol R, Arnheim N (2006). High-resolution recombination patterns in a region of human chromosome 21 measured by sperm typing.. PLoS Genet.

[5] Baudat F, de Massy B (2007). *Cis*- and *trans*-acting elements regulate the mouse Psmb9 meiotic recombination hotspot.. PLoS Genet.

[6] Hey J (2004). What's so hot about recombination hotspots?. PLoS Biol.

[7] Nachman MW (2002). Variation in recombination rate across the genome: evidence and implications.. Curr Opin Genet Dev.

[8] Cirulli ET, Kliman RM, Noor MA (2007). Fine-scale crossover rate heterogeneity in *Drosophila pseudoobscura*.. J Mol Evol.

[9] Tsai CJ, Mets DG, Albrecht MR, Nix P, Chan A (2008). Meiotic crossover number and distribution are regulated by a dosage compensation protein that resembles a condensin subunit.. Genes Dev.

[10] Buchner DA, Trudeau M, George AL, Sprunger LK, Meisler MH (2003). High-resolution mapping of the sodium channel modifier Scnm1 on mouse chromosome 3 and identification of a 1.3-kb recombination hot spot.. Genomics.

[11] Kelmenson PM, Petkov P, Wang X, Higgins DC, Paigen BJ (2005). A torrid zone on mouse chromosome 1 containing a cluster of recombinational hotspots.. Genetics.

[12] Bois PRJ (2007). A highly polymorphic meiotic recombination mouse hotspot exhibits incomplete repair.. Mol Cell Biol.

[13] Nishant KT, Ravishankar H, Rao MR (2004). Characterization of a mouse recombination hot spot locus encoding a novel non-protein-coding RNA.. Mol Cell Biol.

[14] Shiroishi T, Sagai T, Moriwaki K (1993). Hotspots of meiotic recombination in the mouse major histocompatibility complex.. Genetica.

[15] Broman KW, Murray JC, Sheffield VC, White RL, Weber JL (1998). Comprehensive human genetic maps: individual and sex-specific variation in recombination.. Am J Hum Genet.

[16] Donis-Keller H, Green P, Helms C, Cartinhour S, Weiffenbach B (1987). A genetic linkage map of the human genome.. Cell.

[17] Shifman S, Bell JT, Copley RR, Taylor MS, Williams RW (2006). A high-resolution single nucleotide polymorphism genetic map of the mouse genome.. PLoS Biol.

[18] Kong A, Gudbjartsson DF, Sainz J, Jonsdottir GM, Gudjonsson SA (2002). A high-resolution recombination map of the human genome.. Nat Genet.

[19] Lenormand T, Dutheil J (2005). Recombination difference between sexes: a role for haploid selection.. PLoS Biology.

[20] Lercher MJ, Hurst LD (2003). Imprinted chromosomal regions of the human genome have unusually high recombination rates.. Genetics.

[21] Gerton JL, Hawley RS (2005). Homologous chromosome interactions in meiosis: diversity amidst conservation.. Nat Rev Genet.

[22] Petkov PM, Broman KW, Szatkiewicz JP, Paigen K (2007). Crossover interference underlies sex differences in recombination rates.. Trends Genet.

[23] Shiroishi T, Sagai T, Hanzawa N, Gotoh H, Moriwaki K (1991). Genetic control of sex-dependent meiotic recombination in the major histocompatibility complex of the mouse.. EMBO J.

[24] Yandeau-Nelson MD, Nikolau BJ, Schnable PS (2006). Effects of trans-acting genetic modifiers on meiotic recombination across the a1-sh2 interval of maize.. Genetics.

[25] Jeffreys AJ, May CA (2004). Intense and highly localized gene conversion activity in human meiotic crossover hot spots.. Nat Genet.

[26] Jeffreys AJ, Neumann R (2005). Factors influencing recombination frequency and distribution in a human meiotic crossover hotspot.. Hum Mol Genet.

[27] Neumann R, Jeffreys AJ (2006). Polymorphism in the activity of human crossover hotspots independent of local DNA sequence variation.. Hum Mol Genet.

[28] Ptak SE, Hinds DA, Koehler K, Nickel B, Patil N (2005). Fine-scale recombination patterns differ between chimpanzees and humans..

[29] Winckler W, Myers SR, Richter DJ, Onofrio RC, McDonald GJ (2005). Comparison of fine-scale recombination rates in humans and chimpanzees.. Science.

[30] Dietrich WF, Miller J, Steen R, Merchant MA, Damron-Boles D (1996). A comprehensive genetic map of the mouse genome.. Nature.

[31] Rowe LB, Barter ME, Kelmenson JA, Eppig JT (2003). The comprehensive mouse radiation hybrid map densely cross-referenced to the recombination map: a tool to support the sequence assemblies.. Genome Res.

[32] Rhodes M, Straw R, Fernando S, Evans A, Lacey T (1998). A high-resolution microsatellite map of the mouse genome.. Genome Res.

[33] Broman KW, Rowe LB, Churchill GA, Paigen K (2002). Crossover interference in the mouse.. Genetics.

[34] Koehler KE, Cherry JP, Lynn A, Hunt PA, Hassold TJ (2002). Genetic control of mammalian meiotic recombination. I. Variation in exchange frequencies among males from inbred mouse strains.. Genetics.

[35] de Boer E, Stam P, Dietrich AJJ, Pastink A, Heyting C (2006). Two levels of interference in mouse meiotic recombination.. PNAS.

[36] Hunger SP, Galili N, Carroll AJ, Crist WM, Link MP (1991). The t(1;19)(q23;p13) results in consistent fusion of E2A and PBX1 coding sequences in acute lymphoblastic leukemias.. Blood.

[37] Privitera E, Kamps MP, Hayashi Y, Inaba T, Shapiro LH (1992). Different molecular consequences of the 1;19 chromosomal translocation in childhood B-cell precursor acute lymphoblastic leukemia.. Blood.

[38] McVean GA, Myers SR, Hunt S, Deloukas P, Bentley DR (2004). The fine-scale structure of recombination rate variation in the human genome.. Science.

[39] Coop G, Wen X, Ober C, Pritchard JK, Przeworski M (2008). High-resolution mapping of crossovers reveals extensive variation in fine-scale recombination patterns among humans.. Science.

[40] Frazer KA, Eskin E, Kang HM, Bogue MA, Hinds DA (2007). A sequence-based variation map of 8.27 million SNPs in inbred mouse strains.. Nature.

[41] Frazer KA, Ballinger DG, Cox DR, Hinds DA, Stuve LL (2007). A second generation human haplotype map of over 3.1 million SNPs.. Nature.

[42] Holloway K, Lawson VE, Jeffreys AJ (2006). Allelic recombination and de novo deletions in sperm in the human β-globin gene region.. Hum Mol Genet.

[43] Fogel S, Mortimer RK (1971). Recombination in yeast.. Annu Rev Genet.

[44] Tease C, Hulten MA (2004). Inter-sex variation in synaptonemal complex lengths largely determine the different recombination rates in male and female germ cells.. Cytogenet Genome Res.

[45] Kleckner N, Storlazzi A, Zickler D (2003). Coordinate variation in meiotic pachytene SC length and total crossover/chiasma frequency under conditions of constant DNA length.. Trends Genet.

[46] Ooi SL, Henikoff S (2007). Germline histone dynamics and epigenetics.. Curr Opin Cell Biol.

[47] Sato S, Yoshimizu T, Sato E, Matsui Y (2003). Erasure of methylation imprinting of Igf2r during mouse primordial germ-cell development.. Mol Reprod Dev.

[48] de la Casa-Esperon E, Sapienza C (2003). Natural selection and the evolution of genome imprinting.. Annu Rev Genet.

[49] Pardo-Manuel de Villena F, de la Casa-Esperon E, Sapienza C (2000). Natural selection and the function of genome imprinting: beyond the silenced minority.. Trends Genet.

[50] Petes TD (2001). Meiotic recombination hot spots and cold spots.. Nat Rev Genet.

[51] Kauppi L, Jeffreys AJ, Keeney S (2004). Where the crossovers are: recombination distributions in mammals.. Nat Rev Genet.

[52] Myers S, Bottolo L, Freeman C, McVean G, Donnelly P (2005). A fine-scale map of recombination rates and hotspots across the human genome.. Science.

[53] Jeffreys AJ, Ritchie A, Neumann R (2000). High resolution analysis of haplotype diversity and meiotic crossover in the human TAP2 recombination hotspot.. Hum Mol Genet.

[54] Petkov PM, Ding Y, Cassell MA, Zhang W, Wagner G (2004). An efficient SNP system for mouse genome scanning and elucidating strain relationships.. Genome Res.

[55] Myakishev MV, Khripin Y, Hu S, Hamer DH (2001). High-throughput SNP genotyping by allele-specific PCR with universal energy-transfer-labeled primers.. Genome Res.

[56] Storey JDRT (2003). Statistical significance for genome wide studies.. Proc Natl Acad Sci USA.

