# Supplementary material for: The Recombinational Anatomy of a Mouse Chromosome
Source: PLoS Genet. 2008 Jul 11;4(7):e1000119. doi: 10.1371/journal.pgen.1000119 (PMC2440539; doi:10.1371/journal.pgen.1000119)
Supplement: Figure S1 — Graphical representation of recombination, gene deserts, and exon density on Chr 1. (0.07 MB PDF) [file pgen.1000119.s001.pdf]

Fig. S1. Recombination rates, gene deserts and exon density along Chr1. In each panel, from top to bottom: position on Chr1 in Mb, recombination rates (cM) in each of the four backcrosses, recombination rates in females and male, sex-averaged recombination rates, gene deserts larger than 260 kb, gene deserts larger than 1.5 Mb, exon density (bp exons per 100,000 bp window)

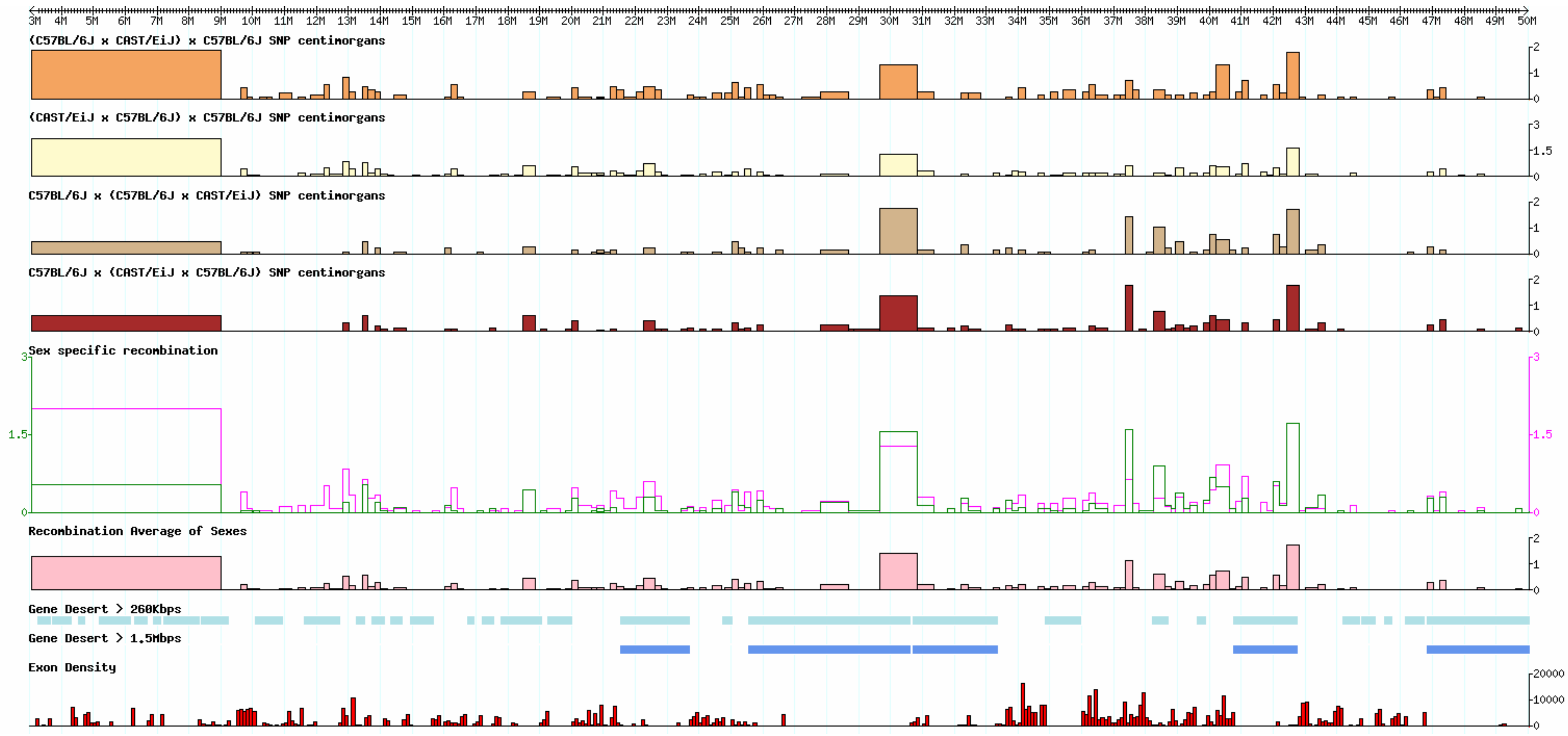

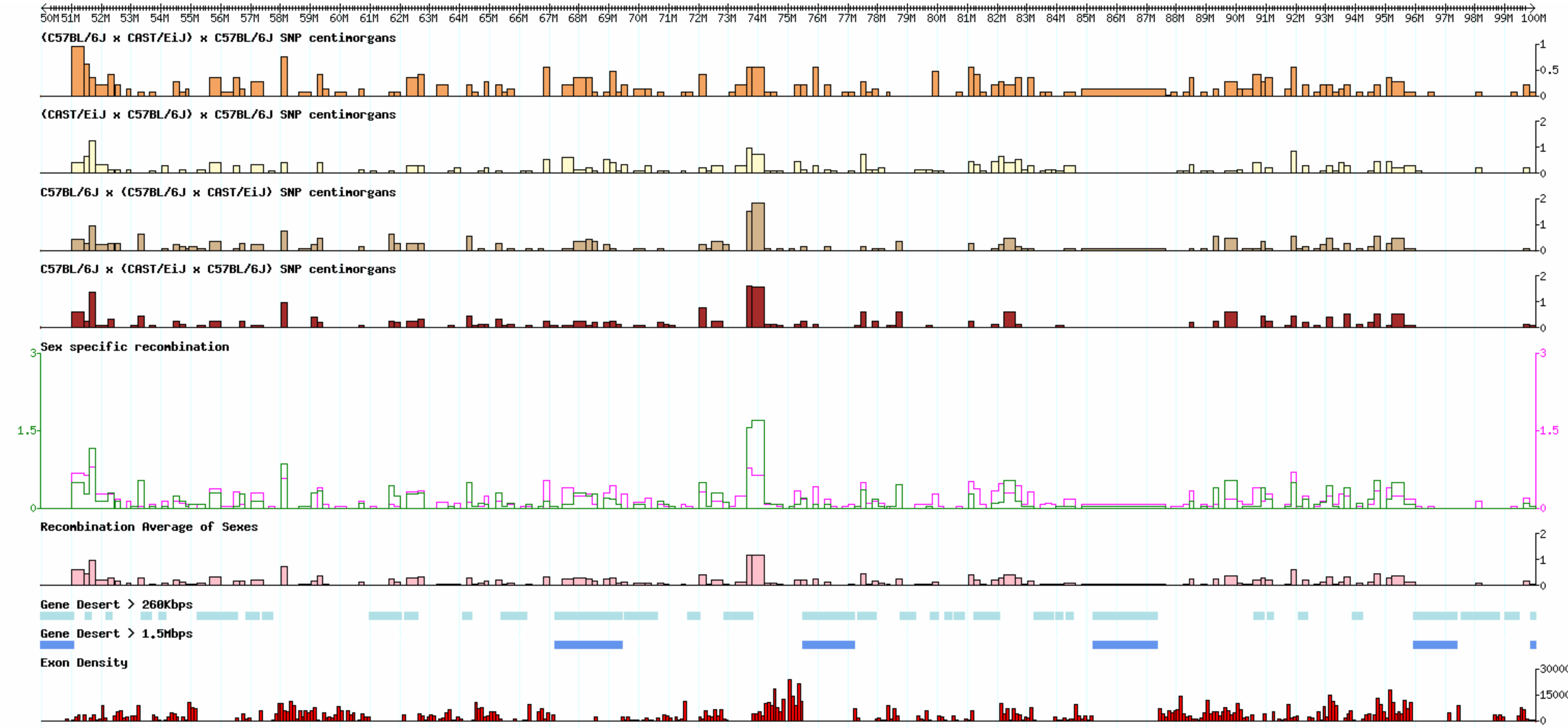

50M 51M 52M 53M 54M 55M 56M 57M 58M 59M 60M 61M 62M 63M 64M 65M 66M 67M 68M 69M 70M 71M 72M 73M 74M 75M 76M 77M 78M 79M 80M 81M 82M 83M 84M 85M 86M 87M 88M 89M 90M 91M 92M 93M 94M 95M 96M 97M 98M 99M 100M
(C57BL/6J x CAST/EiJ) x C57BL/6J SNP centimorgans
(CAST/EiJ x C57BL/6J) x C57BL/6J SNP centimorgans
C57BL/6J x (C57BL/6J x CAST/EiJ) SNP centimorgans
C57BL/6J x (CAST/EiJ x C57BL/6J) SNP centimorgans
Sex specific recombination
Recombination Average of Sexes
Gene Desert > 260Kbps
Gene Desert > 1.5Mbps
Exon Density

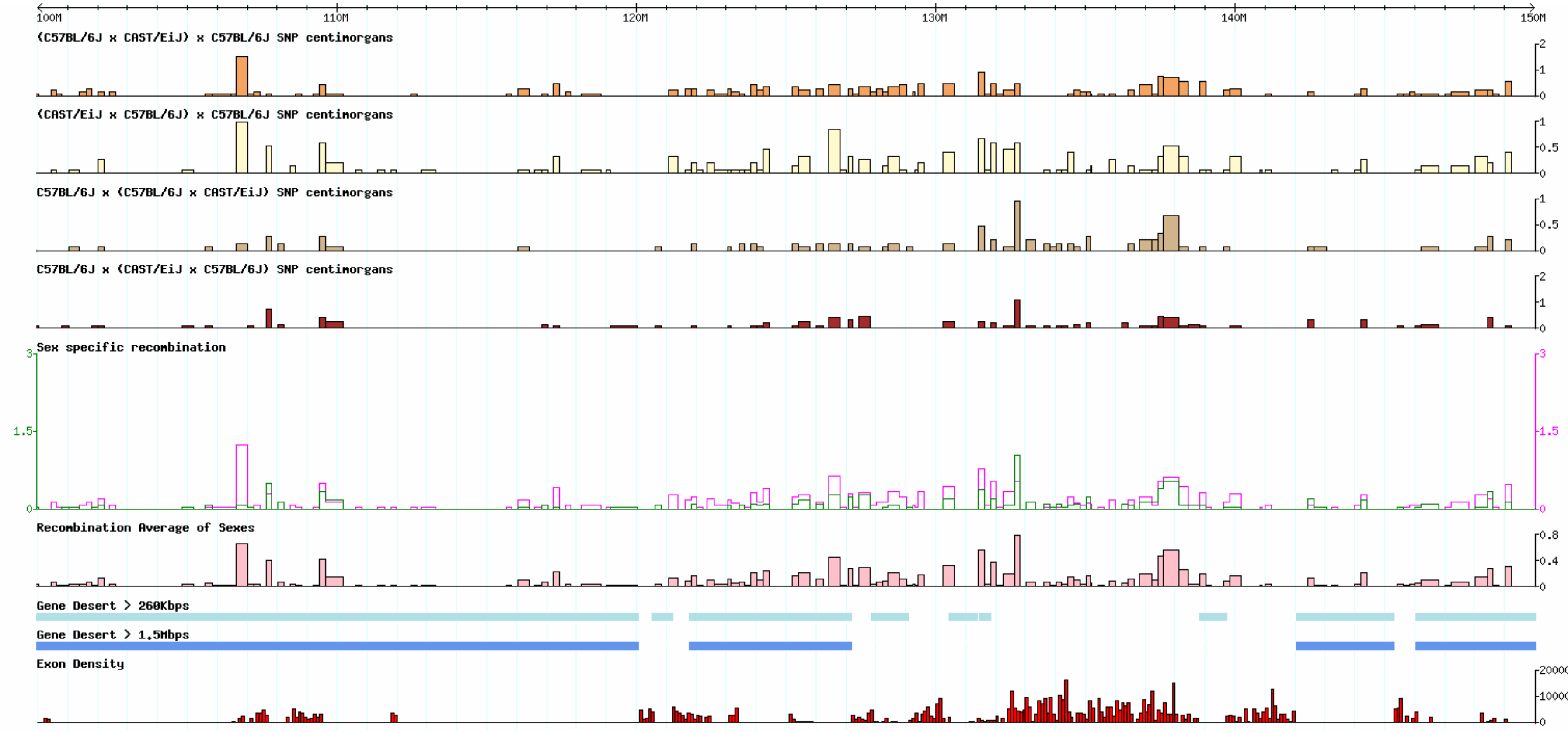

100M
110M
120M
130M
140M
150M
(C57BL/6J x CAST/EiJ) x C57BL/6J SNP centimorgans
(CAST/EiJ x C57BL/6J) x C57BL/6J SNP centimorgans
C57BL/6J x (C57BL/6J x CAST/EiJ) SNP centimorgans
C57BL/6J x (CAST/EiJ x C57BL/6J) SNP centimorgans
Sex specific recombination
Recombination Average of Sexes
Gene Desert > 260Kbps
Gene Desert > 1.5Mbps
Exon Density

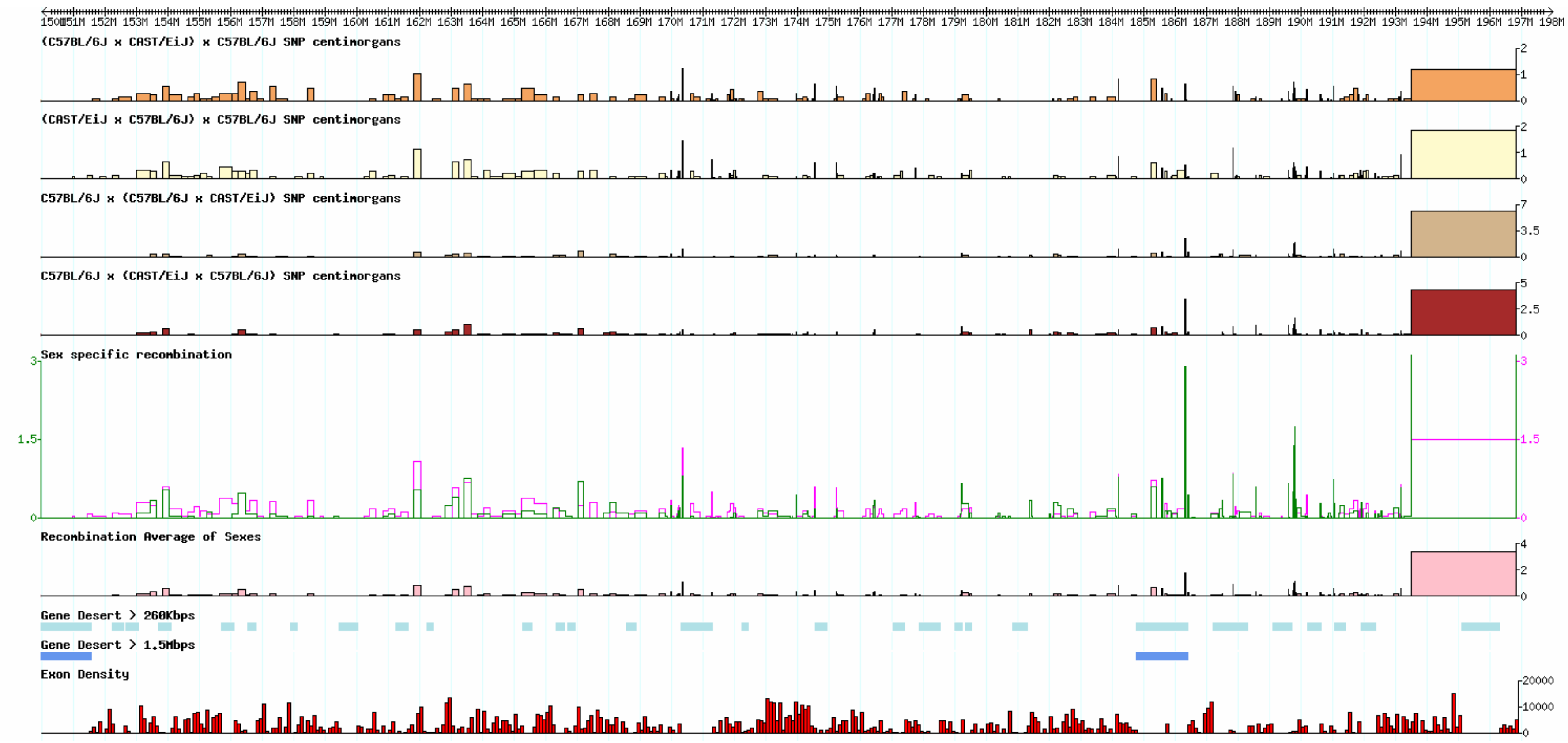

150M 151M 152M 153M 154M 155M 156M 157M 158M 159M 160M 161M 162M 163M 164M 165M 166M 167M 168M 169M 170M 171M 172M 173M 174M 175M 176M 177M 178M 179M 180M 181M 182M 183M 184M 185M 186M 187M 188M 189M 190M 191M 192M 193M 194M 195M 196M 197M 198M
(C57BL/6J x CAST/EiJ) x C57BL/6J SNP centimorgans
(CAST/EiJ x C57BL/6J) x C57BL/6J SNP centimorgans
C57BL/6J x (C57BL/6J x CAST/EiJ) SNP centimorgans
C57BL/6J x (CAST/EiJ x C57BL/6J) SNP centimorgans
Sex specific recombination
Recombination Average of Sexes
Gene Desert > 260Kbps
Gene Desert > 1.5Mbps
Exon Density
