# Supplementary material for: The Recombinational Anatomy of a Mouse Chromosome
Source: PLoS Genet. 2008 Jul 11;4(7):e1000119. doi: 10.1371/journal.pgen.1000119 (PMC2440539; doi:10.1371/journal.pgen.1000119)
Supplement: Figure S3 — Fine mapping of sex-specific recombination activities in the region of 168.8-193.5 on Chr 1 – number of recombinants. (0.01 MB PDF) [file pgen.1000119.s003.pdf]

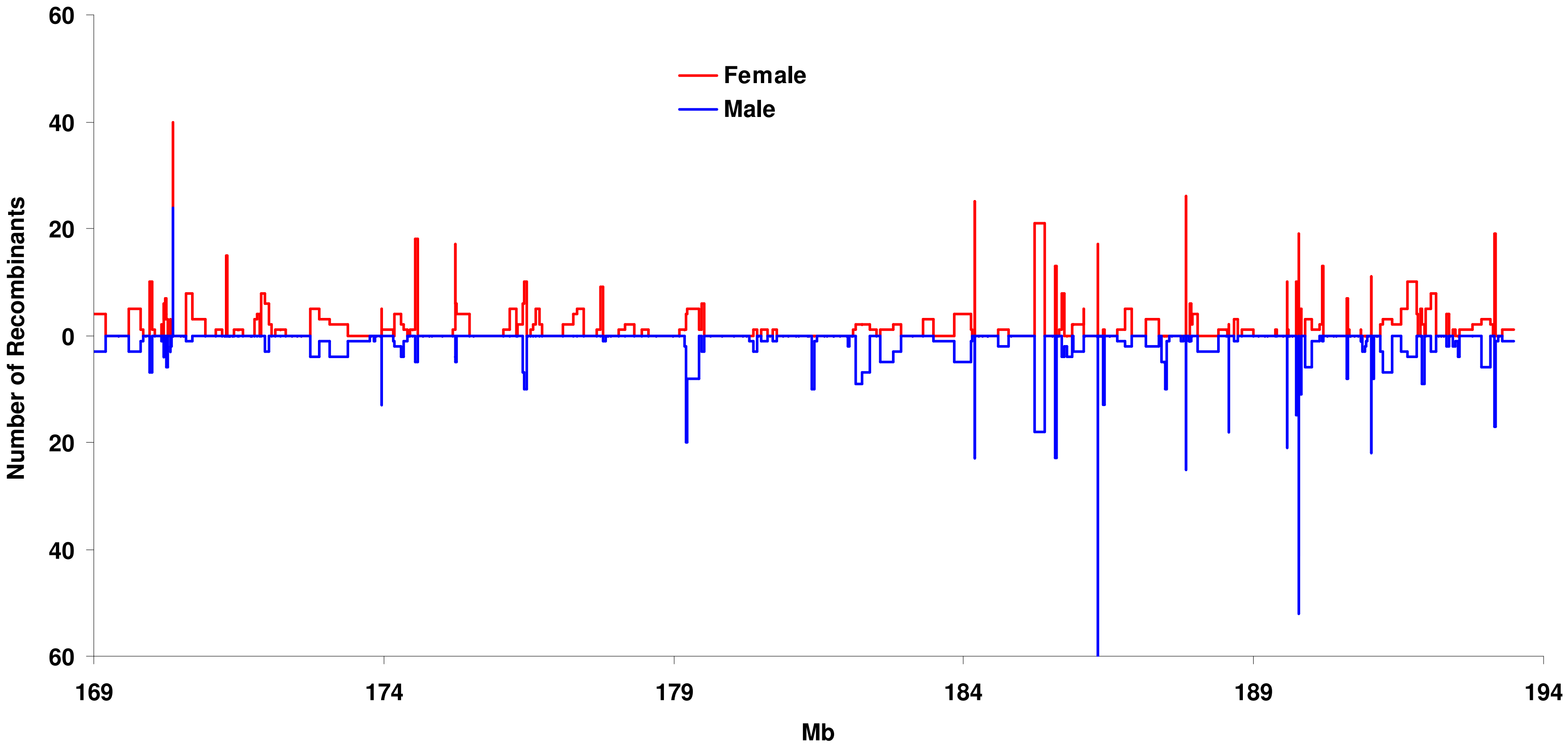

Figure S3. Fine mapping of sex-specific recombination activities in the region of 168.8-193.5 on Chr 1. The number of recombinants in each region is plotted to illustrate the resolution achieved.
