# Supplementary material for: The Recombinational Anatomy of a Mouse Chromosome
Source: PLoS Genet. 2008 Jul 11;4(7):e1000119. doi: 10.1371/journal.pgen.1000119 (PMC2440539; doi:10.1371/journal.pgen.1000119)
Supplement: Table S2 — Correlation between exon density and recombination rates. (0.17 MB DOC) [file pgen.1000119.s005.doc]

Table S2. Correlation between exon density and recombination rates. ***r*** represents correlation coefficient, ***p*** is the probability calculated by bootstrapping. The correlations with ***p*** <0.05 are shown in grey.
