# Supplementary material for: The Recombinational Anatomy of a Mouse Chromosome
Source: PLoS Genet. 2008 Jul 11;4(7):e1000119. doi: 10.1371/journal.pgen.1000119 (PMC2440539; doi:10.1371/journal.pgen.1000119)
Supplement: Table S4 — Approximation of the number of hotspots with given activity. (0.03 MB DOC) [file pgen.1000119.s007.doc]

Table S4. Approximation of the number of hotspots with given activity based on available data.

| Resolution Power (kb) | Minimum Recombination Activity (cM) | | | | |
| --- | --- | --- | --- | --- | --- |
|  | >.016 | >.032 | >.05 | >0.1 | >0.2 |
|  | Number of Hotspots | | | | |
| 5* | 228 | 143 | 99 | 58 | 25 |
| 75 | 163 | 119 | 87 | 54 | 27 |
| 125 | 122 | 98 | 78 | 49 | 27 |
| 204 | 85 | 72 | 64 | 45 | 29 |
| 385 | 54 | 49 | 43 | 38 | 28 |
| 770 | 32 | 32 | 28 | 25 | 23 |

*The numbers are approximated from the available data
