# Supplementary material for: The Recombinational Anatomy of a Mouse Chromosome
Source: PLoS Genet. 2008 Jul 11;4(7):e1000119. doi: 10.1371/journal.pgen.1000119 (PMC2440539; doi:10.1371/journal.pgen.1000119)
Supplement: Table S5 — Direction specificity (imprinting) at hotspots in reciprocal female crosses. (0.06 MB DOC) [file pgen.1000119.s008.doc]

Table S5. Direction specificity (imprinting) at hotspots in reciprocal female crosses. Hotspots with *p*<0.05 are outlined in grey.

|  | Number of Recombinants | | Significance | |
| --- | --- | --- | --- | --- |
| Hotspot Location (Mb) | B6xCAST | CASTxB6 | *p* | *q* |
| 51.6 | 5 | 19 | 0.006 | 1 |
| 138.8 | 8 | 1 | 0.020 | 1 |
| 157.2 | 8 | 1 | 0.020 | 1 |
| 172.7 | 5 | 0 | 0.029 | 1 |
| 37.6 | 5 | 0 | 0.029 | 1 |
| 177.3 | 5 | 0 | 0.029 | 1 |
| 40.2 | 19 | 8 | 0.033 | 1 |
| 79.8 | 7 | 1 | 0.037 | 1 |
| 107.6 | 1 | 8 | 0.039 | 1 |
| 68.8 | 1 | 8 | 0.039 | 1 |
| 126.0 | 4 | 0 | 0.059 | 1 |
| 90.8 | 4 | 0 | 0.059 | 1 |
| 54.4 | 4 | 0 | 0.059 | 1 |
| 128.0 | 4 | 0 | 0.059 | 1 |
| 33.8 | 0 | 5 | 0.062 | 1 |
| 101.6 | 4 | 0 | 0.059 | 1 |
| 191.9 | 0 | 5 | 0.062 | 1 |
| 186.1 | 0 | 5 | 0.062 | 1 |
| 193.2 | 5 | 14 | 0.063 | 1 |
| 136.8 | 6 | 1 | 0.067 | 1 |
| 128.8 | 6 | 1 | 0.067 | 1 |
| 51.0 | 14 | 6 | 0.074 | 1 |
| 187.8 | 8 | 18 | 0.075 | 1 |
| 171.8 | 3 | 0 | 0.120 | 1 |
| 156.2 | 10 | 4 | 0.114 | 1 |
| 77.4 | 4 | 11 | 0.118 | 1 |
| 134.6 | 3 | 0 | 0.120 | 1 |
| 170.3 | 3 | 0 | 0.120 | 1 |
| 10.8 | 3 | 0 | 0.120 | 1 |
| 72.4 | 0 | 4 | 0.125 | 1 |
| 63.2 | 3 | 0 | 0.120 | 1 |
