# Supplementary material for: The Recombinational Anatomy of a Mouse Chromosome
Source: PLoS Genet. 2008 Jul 11;4(7):e1000119. doi: 10.1371/journal.pgen.1000119 (PMC2440539; doi:10.1371/journal.pgen.1000119)
Supplement: Table S6 — Direction specificity (imprinting) at hotspots in reciprocal male crosses. (0.06 MB DOC) [file pgen.1000119.s009.doc]

Table S6. Direction specificity (imprinting) at hotspots in reciprocal male crosses. Hotspots with *p*<0.05 are outlined in grey.

|  | Number of Recombinants | | Significance | |
| --- | --- | --- | --- | --- |
| Hotspot Location (Mb) | B6xCAST | CASTxB6 | *p* | *q* |
| 193.5 | 90 | 67 | 0.017 | 1 |
| 187.4 | 5 | 0 | 0.025 | 1 |
| 187.4 | 5 | 0 | 0.025 | 1 |
| 191.0 | 16 | 6 | 0.030 | 1 |
| 72.0 | 3 | 12 | 0.037 | 1 |
| 188.6 | 4 | 14 | 0.033 | 1 |
| 52.5 | 4 | 0 | 0.052 | 1 |
| 192.5 | 4 | 0 | 0.052 | 1 |
| 42.2 | 4 | 0 | 0.052 | 1 |
| 68.2 | 6 | 1 | 0.060 | 1 |
| 189.8 | 26 | 15 | 0.057 | 1 |
| 144.2 | 0 | 5 | 0.064 | 1 |
| 170.0 | 6 | 1 | 0.060 | 1 |
| 170.3 | 16 | 8 | 0.068 | 1 |
| 127.4 | 1 | 7 | 0.072 | 1 |
| 77.4 | 2 | 9 | 0.068 | 1 |
| 190.6 | 1 | 7 | 0.072 | 1 |
| 163.4 | 7 | 16 | 0.141 | 1 |
| 82.1 | 3 | 0 | 0.109 | 1 |
| 188.0 | 3 | 0 | 0.109 | 1 |
| 92.8 | 3 | 0 | 0.109 | 1 |
| 72.8 | 3 | 0 | 0.109 | 1 |
| 155.2 | 3 | 0 | 0.109 | 1 |
| 189.6 | 6 | 14 | 0.121 | 1 |
| 174.3 | 0 | 4 | 0.126 | 1 |
| 107.6 | 4 | 11 | 0.123 | 1 |
| 66.8 | 0 | 4 | 0.126 | 1 |
| 185.7 | 0 | 4 | 0.126 | 1 |
