# Supplementary material for: The Recombinational Anatomy of a Mouse Chromosome
Source: PLoS Genet. 2008 Jul 11;4(7):e1000119. doi: 10.1371/journal.pgen.1000119 (PMC2440539; doi:10.1371/journal.pgen.1000119)
Supplement: Dataset S1 — Sequences of the newly identified hotspots with SNPs between C57BL/6J and CAST/EiJ. (0.07 MB DOC) [file pgen.1000119.s010.doc]

**Sequences of the newly identified hotspots with SNPs between C57BL/6J and CAST/EiJ.** The regions where recombination events were detected are outlined in yellow. SNPs present in the NIEHS/Perlegen database [1] are in red, SNPs we identified are in green. The sequence positions are according to NCBI Build 36.

1:186316101-186319301

ACTCTTGTT[C/A]CTCCACAACAGCTACCTACTAAAGCCGGGAGGCCAGTGGTCCTTGGCCTG

TTTCAGGCTCC[G/A]TTTTCATATCTCACCTTAAAATTCATTCCCTACAATGGAAGAC[-/A]AAAAA

CTTAGTTATATTATAATTCATCCCTAGCAAATACTCACTTCCCCTGTCTCT[-/TACTTCTAA]ACATTTTTG

ATGAACCATGCTTGGTTAAATTAATG[T/C]TCGATGCTTGTGGGGCTGGAGAGATGACTCAGA

GGCTAAGAGCTCACGATGCTCTTCCAGAGGACCCCGGTTTCAGTTTCCAGAG[C/T]CCACACC

AAGTGGCTCACAACTGCCTGTAACTCCAGCTCCAGAGAATTGCTCTGACCGCCTCAG[G/A]CA

CCTGCATATACGTGGAACACCTAACATAGACAAAAATAAAAATGCAAGTAAGTATTTTT[T/A]

AAAATACAGTGATTATACCCTTGTTATATAAACGTCACTTATAGATGACTTAT[G/A]TAATTT

[C/A][A/T]AATTGTA[T/A]TTTTTAATTATTTCTTTTTTAATTATCATACAAAGTAACATGTTTCCTTGA[A/G]ATGTTTTCAAATAGCCTTTGTGTTGAAACCCTTAAGTTCCTCTCCATCATCTCCCTAC

TCCCTCTCCCAGCTTGTCCCCTCTACCTTCAGTATTTTCTTTCTCTTTTTCATGTCACAT

GTGAAGGGACATAGGGAAGGGGAATTGTATTATGAAAGAGAGGCCACCAAGACTCAAGAC

CCCTGGAAGTACTTTAAGACACAGAAGTCTTCAAGCAAGCTGTGACCTGAGACATTGGCT

GCTCTTTAAAACAGTGGAAGTCGTCATTGAAACACCATTTAGGTAATGGAGACACAAATA

GAAACAACTACCAAGAACTTCCCATCAGCAGTGAAAGGGTCACTTCCTTTTTTTTTTTTT

TTTTTTTTTTTGGTTAAATGCCCACCCTGAACACCACCACTCCTTTCCAGGCAGAATTCT

GTCGATAACCCATTCCAAGCAAGACTCTTGAACTTCCACAGCCCTAGGGTTTTCTTTGGA

GGGAAAGTATAAGGATGTTTTGTCCTACCTCGGGTTGCTGGCTTCTTAAATTGATCCTTG

CTACCCTCCCCCTGCTCATTAGCCTTGCATGTCCTGACCTACTCTCTTCTTTCTTTCCTT

TTTCCTCTATTTCCCCAAATCATTACCCTTACATACCTAAAAACTGCACTAGT[T/C]CCTAGC

CCTTCCTTATCACAGCATACATTCCTTACTGTTTGGGTATAAGGTATCCAACCACCCAGA

TCATCTTTGCTTGAAATACTAACTACATAGAGCTGTAAGCTTCAACAGTATCAAAGCCTG

ACCCTCATTTGGCAGGGCTCCAGAGAAGCAGGGACACTGTCAGGGAAACTGGTTTGCACC

TTACTTATTTCTTCTCTCTGCCCAGGCTATCTAGGTAC[G/A]TTGAGTTTGGGCTTTTGTTTA

GTTGTTATTGTATGTGTATGAGTGTTTGTG[A/C]GGTTATATGTGTATAATCATCTGCATGTT

GTGCCCTTGGTGACCAGAAGAGGGTGTTCTATCCTCCAAAACTGGAGGCAGAGTCAGTTG

CAAACCACCATGTGGGTGCTGAGAGTTGAACCTGGGTCTTCTGCAAGATCAGTAAATGCT

CTTAACCGACCAGAGCCTGTACACTGATGTGGGAGGAGATGGTGGGTGAATA[-/A]GTGT[G/T]CAG

ACTTGGAC[C/T]CTGCCCTT[T/C]CTTTACGCATTCCACTAACCATGGGATGGAAGGTTCTGCCTC

TCAAAACCTTCAGTTCTGCATCTGTGAAATCATATTA[G/A]CAACCTTGAGTCTAAGTGGTCG

GTGTGAGTATTAGACGTATATGCTC[G/A]GTTTATTAACTTCAGCTTCACGTCAG[A/G]GTAGGAG

ATGTATAAAGGGAAAAACAAAACAGAGCATAAGGTATAGTAGCCTCGGGCAGCCATCTTC

CAGTAACTCCCCAAAATGATGAACACAAAGGGAAAGAGGAGAGGCACCTGGTATATGTTC

TCTAGGCCTTTTAGGAAACATGGCATTGTTCCTTTGGCCACATACATGGGAATCTACAAG

AAGGGTGATATTGTGAACATCAAGGGAATGGGTACTGTTCAAAAAGGAATGCCCCATAAG

TGCTACCACGGCAAAACC[G/A]GAAGAGTCTACAATGTCACCCAGCATCCCGTAGGTATCATG

GTAAACAAGCAAGTTAAGGGCAAGATTCTGGCCAAGAGGACCAGTGTGTGGACTGAGCAC

ATCAAGCACTCAAAGAGCAGAGACAGCTTCCTGAAGCGGGTGAAGGAGAACGATCAGAAG

AAAAAGGAAGCCAAAGAGAAGGGCACCTGGGTGCAGCTGAAGCGCCAGCCTGCGCCACCC

AGAGAAGCACACTTTGTGAGGACTAACGA[T/A]AAAGAGCCTGAGCTGTTGGAGCCCATTCCA

TAC[G/A]AATTCATGGCCTAATGTACAAAAAT[G/A]AAATAAAGGACCAGGACTGGAAAAAAAAAA

AGGTATAGTAGCCTCAATAGACACGAGCTGCCTTTGCCACTCTTGATTAACTCCCACAGC

CTTGCTCCACTGACCTGCTAGTTGCCATGTCTCTT[T/G]GTTGGCTGGTACCCGTTCGTTTA[T/C]

CACTTGATCTGCCTGCAGGGCTTGAGACTCTCAAA[C/T]CTTCTTCCCAGCCGACCAGCACCC TGGCCTCATCTCAGTGACCCAATCCTGTTTCCTAATGAGCCATTTGTCCCTCGAAATGAT

TCAATCAATGGAGTAAGAACTCCGCCATGATTAGGAAAGCTGAGGGAAATGGGCAACAGG

ACATGGC[A/G]CAGTGACATTCACATTCACATT[T/C]AGAACCATAAAAATGTGGTAACTGGTTCT

GCCTTTTGTTCCATCCAGTTGTCTCATTTTGAAACTTTCCTGGAAGAGACTG[A/G]GTATTAC

TGGACGCTATGATGAGGTAGACATTTTTTTGTTGTTGTTGCCCAATCACATGAGAATCAT

GTAGAACGGACA[G/A]CCAGTGAGGAAATGATGTCATTCTTGAGACAAAGAGAGGAAGTTGCT

GAGAGAGCATGGGGGAGGGGAGGGGTACTTCAAACACCCATCAGTGAATCATTATGCGTT

TAATAGCTTGAGCCTCTAGGTTACCAATTCTTTCTCTCACAGTAATATCTAGCTCCTAAC

TTCCAAATCCTTGATCAAGTGGATTTGTTGCATATACAGATTACACAGTGATCTGGGACA

ATGAACACACATAAGCAATGT

1:187827000-187831000

ACACACACACACACATACACACACACACACACCTGCACAGGTACACACACAGGCATATAC

AAAATAAAATTAAATAAAGAACTACTATGTGATCAAACTGTACAACTGCTGAGCATATAT

AGCCAAAGGAATCAAAGTTTGTATATACACGCCTGCATGCCCATGTTTGCCACAAAAACC

AAACTATAGCATCAAAATAGGTGCCTAAGTATAGACATCTGCCTGTGTTCACATTTGGCC

GATGTACACAATGGGACATTGAGACGCAGTGAGGAATTAAAGGACGTGGTTTCCAGGGAA

ATGGAGAGAGCTGGAGTTCACCCTGTCAAGGGAAATAAAGTAAATCTGGGGAGACAAGCA

TCATATGTTTCCTTTTATAGGGAAAGCCTGAAACAAATGACACAGCCTTCGAAAGAGGCT

ACTAGAGAAGAAAGGGGATCAGGTGAGGGTAGGGACGGTAAGAGGGAGGGGGAGGGAATA

TGACCAGAGTTTATTTTATGCATGGATGTGAACCATCTGATGAGAACTAGCATTTTCTAC

ATTTACTATAGTGTGTGTGAGTGTGTGTGTGTTTTAAGTAAGAATAAAACTGAGGTTTTT

CTCCTTTTAATGAGTGGTCTCTCTTGGGGGGTGATAGGCTGTCCTTTATGGTCAGAGATT

GTGAGGGTAATGGCAGTTATTTTCGAATCTGAAGACAGAACTGTAGTCTGTTTTTCTGGC

CTTTGCTTC[T/C]AAGGACACTATTGGTTTTAATTACTGGGTTTAGTTTAGTTTAGTTTAGCT

TTTTTAGCTTCTG[G/T]TTAACTAAATCCCTTCCTCACAGATATCCCTTCATTGCCCATTGTG

AAACGGCTGAGGCTCCTGTCTTACACACCTGACCCCTCACCTCTGGCCCCTCCCCCCATG

GCCAAGCCTTCCAATAATCCCCCCCAGCAGTCCCCCCATGCCATTGTTTGAGGAAGGAGC

TAATGCTCTTCCCCCACCTCTTGCTGCCAACGGTCAGTGGAATTGTGTAGCTATGAATGT

CACCTGAT[T/G]TCAAATGGGGGAAAATCACCATGACAGTGCTTCAACATTGGCACTTGCACA

GCATAACCAGAAATGCAAAATTTGCCCGTATTTACCATCTTCTAAAGTTGGATAGTCCAA

GGGTCAAAACACAGCTCATTAACCATTCCTTCCAGCAGAAAGTTGGGATCTGGCATTAGT

GTTCTAGAGATGATGTGGAAACCAGGACAAGTAGCTTTCCTGTTGTCTCTCAATTGTTCT

GTATTTCCACTGCATAACTAACCTTAGTTATCCTTACCTGAAGATTGGTTAAACTTAAAT

CAAGTTGGTTAAGCTCTTAGTAGAATGGGCTGCTTCCCAATTATGTTCTATGTTGTATGT

TAAGTATAATTGA[A/G]AAACATGCACTCTTAAAGGACTTCTATTCTTT[A/-]AAATTTTTTTTTC

TTTATAGTGTAGAGAAAGCTAAGGGAGAGTCACTATC[C/T]ATTGGGGATCAGGCCATGAGAA

G[G/A]TGGTCCTATGGATGGCCCCACACCCCCATGGGCATATGAACAGCACCAGTCTGACTCA

AGGAGAATGGGAAGTTGGGAGGATGCT[G/C]GGGTTAGTGGTCCCGGGAGGA[T/G]ATGGAGGAGG

ATAATAGGGGATGGATATGATCAGAATAATTTGTATAAATCTATGAAAGAAGAGATGATG

GATAAAAATGCTATAAACAAGAAAAGAAAAGAAACTGCAG[A/G]ATGATAAAAAGGTAAAAGT

TGTATTATCTGCTTAATAGTTGGAAATTATTACAGACACCAAAATTGTTAAGTTTAGCAT

TTCTAGGGTATTTTATGCAACATTAAAAATATAGTAGTGAGAATAGATTTGTTTCTGAGC

AGCATCTACCATATGCAGGACTTGCATCTGTATTTGTGGAGAAAGTGGTCCAGCAATGCA

TGTTATTAAATTAGAGAGAGAGATAACAAGAGAGACAGTGTCACACAGTTCACATCACTG

TCATCTTCAGTTGCACACTCAGACACCTCTGGGAACTTAACAAGGGTC[G/A]CTGTGCAGAGG

TTTCTTAATATAATTTGTTATACTCAAACCTCTGGCA[A/G]TTAGAGTGTCTTTTAGAAGTAT

CCCCACTATGGAACAGGAATCCTCACAGGATTTGGACTACCCAAATGGTCTTCCACCCAA

ATGCTGGTCACATCTGTCAAATTTTCCACTACACATCTCAGTAATAAGTATTGCTATGGC

ATAATGAATATTTGGTGATGCATTTCTTGAATCCATTGAAAACTTTACTCAATCAATGAA

AATATAATCTCCACTATTCCAATGACTGGTGTTAAGTTCTGTTGGTGAGAGACCAGAAGT

TATGGAGTTTATCCTCAACAGGGTGATAAGTGGTAAGAAAAACAGCTC[A/G]GTGGGAGTAAT

AAACACTAAAGGATGGGACAGGGCAAGTTCTAGAGGTTCTAGGAGAAACACTTTGCTTGT

TGCCATGTAATATCAGAGAAGGGTCTGATGCAATAAA[T/C]ACCACAGTCCTCAGAATCCATG

CT[A/G]TAC[T/C]AGAAGTTCTCAGACAATGGGGTCTCAGAGATGAGGGCTGTCCACCT[T/C]CCCCTTTTCCACTGAAGCTTGGTGTTTCTCTATGGCA[G/A]ACCAATGTGCCTAGCAATACACTCTGGT

GCCTCTCTTTGCCTCTTTTCCTTATTCTGGCTAAGTCTCATTTTATCCCAAGCCCAAGAA

TAAAGCAGTACCTTCCAT[A/G]ATTAACTGGTGAGATCTTCCTATGATAATGGGATTGTTCTC

TTGGATTTATTAAGCATGTGTGGTGTAGAGATTGTCCACTTGTCCTAGATGTAAGATGAC

TGGGTACTGAACGGCTGACTCGA[G/A]AGGGACTGAATGTGTTGAGTGGCCATCAGCTAGGAA

CCATAGCAGCCAAGTCAAACAGCGAGTACCAAA[C/T]GTACAACTTCAGGTCAGTTAAGGAGT

GTTCTCAGCACAGTTATTTGAGTGTT[G/A]AGAAGCTGAACTCAATGTTCCTTGGAAGGCTCA

AGCCCCGTACTTGGGTGCCAATATTTAGATAATTAGAGATCAGTCACCGTGTGGAAGGGA

CAGTCCTAGTTCTTTATGGATAAATAGAACATTGTGGA[G/A]CGTTTCCAACAACTTTCCTAG

AAACGACCCCTCTGAAGTTGAAAACAGCAATGAAAAAGTGATCCC[G/A]TTTTATTAAGGCCT

TACCACTTCTGACTTCTTAGACG[G/A]CTAGTCCACAAAATGAAAA[-/G]AAAAAAAAAAAATCCCA

CGCCATAGCTCTCTGCGCCTTGTAAGTCTGTTACCGTGACGGTTCTCTCTCACAGTATCT

GCATCTCGATGTCTGTAAGCATGGGATGGGTTAAAAGACCAGTCTCTGAGATGCTATGAG

GCAGAACTTTTACTTTTCAGCACTGATTAGCTCAGCTCCCCAGCTGTGCAGCACCACGCC

CTGGGGTGCAACTGAGAGCGAGCCCCCAGACAGGTTTATATTGGCTTAAATTTGTAGCTT

TTATTCGGGAAGTTTTTATAAATTACATTGTGATGTT[G/A]TGACTTTGCCATTATAGCCTGT

ACAATACAGAGCAGTAACTCAAGGGAATGTTTGGCTCCTTTAAAGAAATGTTAACAGCAA

AGAGAGTCGAGGTGGAACT[G/C]AAGCAGGAAGTGAATTAACTAATTACCTCATGAGTGAGTT

CACTGACTCCAATTCCTGAATATATCAATATATAGCCTGAAAAAATATGTTCAAAGTTGT

ATGCCAGTAGATAAAAATGCAACAGAGTCAAACTCCGCTGAACTTTTTGACACACTTATT

CCTATAATCGCCTCAGAACTCCATGCCAGTTTTCAAACAGGAAGAGCAAGTTCAAAAGGA

CAAAACAAGGTTGGTGTCACAGGCATCAGAGAGAAAGTGGTTAAACAGTTATCGTGGTGT

GAGACCTTTCAATAACTGATGACAAGTTTACAATTTTAAGATAAAAATTGAATCCTTGGC

TTCTTCACTGATTTTAAGCACACCCTAAAATGTGGGAAACA

1:188580000-188584000

GGATGCAGGTACGGCAGAGAGGCAGCTGTTGAGGAGAAGGAAGTAACTAGTGATGTGCCC

TTGTGGGAAGGTGCAGTGAGAGCTGAGGACCCCTGAGGAGCATGAGTTCATGTTGGTGC[C/T]

CCTTCAGACCGATTGGCTGACTGATTGCGGCTGTGGAGTGCTGTCATATGACAGGTTGAG

AGCCAGCATTATCTTAGGCTACCTTCTTAACAGCCATTTACCATCAGCCTTTGCATCTAA

CCTAGCATCTTTTGGACTATCGGGAAAACCTTTAGGATATACTCAGCTAGTAGACCACTG

GCTTCTACAAACTCCAACACTGAGGGCACCCTCAGATAGTATCTGAAACCTGAGCAGAGG

TTTCCCCACATTGCCTGTAACCCGCCTCTCTGACGCCCTTTTCAGAGGTATTTGGTAAAC

TTACTGATTTATTCTATGAGGGTAAAAGTTCACGTTGGACCAGGAGATGCCTCCAAGGAC

AAAAGCCCTTGCTGCAAAGGCTGGTG[A/G]CCTGAAATCAGTCACTGAGCAAGGAGAGGAC[C/T]G

TCTCCTACAAGCTGTCCTCAACCTCCACATGTACACTGTGACA[-/A]CACACGCTCATGCATG

AACATGCATGGGCGAACACGCATGCATGCACACACAGAGAGAAAGAGAGAGAAAAATAGT

TTTGCTTTTTGTTTTGTTTTTGTTTTTGTTTTTATGTAGCCATTTCTGCTCTGAAACATG

GAACCCAGAGCAATCTGAGTCTCTATCCCAAAGTCATGATTTGGCT[C/A]AACATAAC[A/G]GACT

CGTATTTTCTTTGAAGCAAGACCTGTGCTTTACCTTAGTCTT[A/C]AGAACTGAGCAC[C/T]GGGA

TTGGCTGAGTGCTGCTCTGTGTTCAAATGCTAAATTCCATACCC[T/C]GAGATCTGCTGGCCA

CAGGATGAGTGGGCTCTCACACACACCAGCTTTTGTTGT[G/A]CAAGCCTTGCTCCCCAAGTT

ATTGGTCAACAAAAGGCTAAGCCTGTGATTGTTTACTAGAGAGAGGAAGGCAGGACTTCC

TGTGAGGGAGAAAGGAACTCTGGAAAAGAAAAAGAGGAAGGCCTTTCCCCAGGAGACATG

AGACAGACCTAACTA[T/C]AGGCCTGGAAGGTATAGTTAGCCAAGTGGGCTGGATGGAGCCAG

GTGAGAGTCTGTCTAGCTAAAGCCTAAGCTTTGAAATATGATTAAAAGGTGTCTGTATGG

TTATTTGGTAAACAAGCTGATTAAGGAATAACTACTGCTGCTATATCACTTTGCAGTATT

AATTAATGTATGCAGAATATTTGTAATCATTTTACAACTGATACTAGATTGCTGGTGACA

GTGGGTTGGTGTGAATCAGTGGAGGATACTATGTGGTAGTTGAGTGGATGCCAAGGTGCT

TATTAA[A/G]CGGATCACTGCTTGTATTAACTGCTTAGTGAAAGGCTGGAGGAAGGAAAATGG

AGTTAGGAAAACAGAAACGCTGAGCAATTCTCTTGACCAAGAATGCAAACTTCATATTGC

TGTTACTCTGAGTCTCTGTGATGGTGACTGGGATGTGGCTGTTCTCTGAGA[T/C]CTGTGACA

GACTTTGTGACAGAAATGAGGTCTCCTTCCTGGAAGCTCACCTTGCT[T/C]CCAACATCAGTG

C[G/A]GACCAGGAGACCCATGACAGC[C/T]CCAGG[T/A]ACTG[A/T]GATGCCCCTCGCCCAGCATGCTTATGGGCAGTGAGGCCATGGTAATTCCTGTCCC[C/T]CTGACAA[C/T]CCACAGTGAAAATATAC[-/T][-/A]TGTCTTTCCTTTCTGTGCCAGAGCAAAAATTAAATATAATTTCCACTTTATTCCC

TTTGTACATGTGTGCAAGCATTGCTGTTCTCCACGTATAAAGAAAAAGTAAAAGTGTAGCAGGATGATT

AACTGTAGGACTGTTATTCAGTTCAGCCCATTCTCTCTGTGTGTGCGTATGCACATGTGT

ACATACATGTTCACGTGTGTTCATATGTGTACTTAGGTTTTTACTGTCTAAGCTACCTCC

CCAGCTCCTTCAACCTATTCTTAACTCAGGCTTGATTCTTCTCGAACAGTTTTCAACTTG

AGCCTCAGCA[T/C]TTTTTGAAAGTACCAAACTAATGAAAATAAAATTATTTCATGTAGAGAT

TATAATTTAGTAGCTTTTGTCGGCCCTCCCCTCTACAGTACATGACATTTCTTG[A/T]ATTTT

GGAACCAAGCTTTCACCGAGGGAGGGTGTATTGTATGTAGGGTTAGGCAGTGCCAATGAC

CGATCTGACCAGTTTTTGCCAACGTAATCACCAACTGACTCTTTTCAAGAACCCCAGGAA

GTAACCAACTTCTTTCAATCGCTGTCACAAAATCTTAATGGTCCCTTCAAACAAAAATGT

ACCTCGCAGGCCTAGTAACTGACCTATTTGATTCCAACATAGATCATAATAATTTACCAG

TGTCGCTCA[T/C]TCGAGAGTCTGATTGCCTTCTTCGCCTCCGGGTGCTGGAAGAAAAGGGCT

CTGCGGCTGAAAGGGTTAAGTGGATTTTATTATAATGTGTCCCTGTTTCATTCGCTCCCT

TCAGCTTCTTCACAAAATGTTAATCTAATTGTGATACTATCAGTTTGATGACAATATGAC

AAGACAGACAAATTTGATTTTATTCTCCACAGAGCAAAGCATATGCTCTGTGAAGGCCTT

GACAGAGAGAAGGGCATAAAACAAATGGAGTTCATGAATATACAATTTTTATGGTACACA

ATAGTGACATCTAGACTTCAGAGACTAGGTGGGCGAGCCTGAGACTTTACTATTGAGATT

AATGGGTACGTAAAAAGGTGCCAACAGAGAGTACTACAGTTTATGTTAAAAATGTCGATG

TGGATAAGAATAATTTTCTTCTCAAGATGGATGATACACTTAATGGCTGGGAGACATTTG

GGCTCAGTAGCAGTGCCTCCAAAGTAATTTTTTGTTGTTATTGTAAATTATATAATCTTA

ACTGCAATTATAGTTCTGCTCCAGTAAAGGTCAACTTAGCACCGTGGATGGTTATGAAGC

AAAAATGGAAATTCGCATATGGATTCCCTTGTTGATCGCTTATGAATGATTTCAAAGTAA

GATGATTTTTTTGGTAACTCTATTTCTCATTTAATGCAATACAGCAAGTAAAGACAGCAC

ACATTAACAAAACACAAATTAGATTTCTCTAAGTAAACATGCATCTTCTAATGGACCAGG

GCTGGTGGAGAAAGGACAGAATGAAGGTTAAAGACACCAGGCGAATGGGAAAGGTTTATG

TTGAAGAAAATCAGAAGCAGAAAGTATTGAAGAATCGTTGATGGATTTAAATCTCTGCTT

AGGTTTGGTTTCCAGAACACAAAGTCCAACATAG[C/A]TGCAGCCTTTGGCTCTCTAAAATTT

ACTCTGATATTGAGACTCAGCTCATCATTTTTGAAAGGCATGCCAATGATTGTCTCAATA

CCTGGTTGCTAAGCATCGGTTTCTGATCTTCCTGTGGTGATGAG[C/T]TTGAAGGGATTCAAT

CCAGGCTAAATTTTTAATCCCAATCTTTTTCTTTATTTCACAAAGAAATGCAAATGCCAA

GCTCACAATATGAAATATGTGACTGTAGAACATACCAGACTTTAAAGATTTCTTTATTTT

AATATAGATAGTTCCTGAAATCAAAACTCTACAACAATATACTTAGAATTAATTGTGGCA

ATGTATCTTGAGAGCGTTGAGTTTTAATTATGTAACTTTTATCTACTAGAGCTTTCTCAT

GGTTCTGATCTACAGGAAAGTTTACAGGAAACATTAATAAATTAAAATGGCCAGAGTGAC

CTTTATACTTTAAAGATCAGCAATGATTATAATCACAGTTTGTCCTGCAAGTTGCCAGGC

CCAGCGTCTCTCCCATGTACCACTCCTTGCTCATTTTCAAATTCATGGTCTCTTTTTGTA

ATTAATTGTTATTACATGTGCATATATGTGTATATATAATGTTCCTAAATACATAAATAC

AACTTGCTCCATCTGTATAATGTAACTTGTATCTTTATGTT

1:189583000-189586000

GCTGGAGGTCAGAAGTCCAAGTCATCCTCAGCTCCATAGCCAGTTTGTGGTTAGCCTGGG

ATGTAGGGGGTGGGGCTGAGGAGTTGGGGAGAAGAGGATGAGGAGGAAGAAAAGGAATAA

GGACAAAAGAGGATGCTTACCCTGAAGTCACCTCTGTTTTGTGCCCCAAGAGCTTCTAAA

GGGAAATCATCCAGGGAAGTGAGAAGATCCGTGCTAAGAACCCAGCCCCGTGCCTGGAGT

ATGGAGCATGCTCGTTCATGTTCTCTCTCTCTCTCTCTCTCTCTCTCTCTCTCTCTCTCC

ATCCATCCACCCCCCCTCTCCTTTTCCCTCTGCTTCTTTTCTGTCCTGCCTTTCCTTTCT

CAAAAATAATAATAAAAAAACACTTCCTATTTTCAAAATGCCACCATATTCTGTAACAAA

GTACGCAGTCAATCTGTGGTCTTGGTGGCAACTGTGTAACAGGCACTGCATCTGTCAGCA

AGTCGGCCTCAGTAAGCTGGCCTTCTCATGCTAAGGTGAGTGGTCAGAGAGTGTGCAGGC

AAGGGATAAGCTCACAACCTCACCCTCTAGGCTTGTTTTTTCGAGTTTCTGTACAGATCT

AGTGCTTCACTGCAGGTGTGGTACAGGTCTGCTATCTGTTCTGCCTTCTGCCCAGATGAG

AAGAGAGGGTCAAGGGTTTCAAGCCATCCAAACTGAGGAGGAGTTGAGAAAAGGCAAGGA

TGCTAGTTGCCATCTCTTTGTGTCCAAAGATACTCTGATGCAGAGAAAACACATCATTCC

AGGTGCTGCGAGGCAGCTGCTTTCATGTGGCCAAAATCAGAGCTAAGTTTTAGTCAAGGA

TGCAGAGATGATCCTGTGCAAGTGAACAGGTATCTGTGTAGCCCAAACTCCCCTCTGGGT

CCCTGGCTTCAAGTTACTTACCAGGAGG[C/A]AGTAAGGGATATCACCTGGTAGGAACAGAAC

TAGCCTACCACAGTTCACCACTACACTCCCTCTCCAGCAATCTATACAATGAGAAGGTCA

TATGGCTGAAGATACTGGCCGCTCACACCTGGCTAGGGGCAAAGAGACTCAAGGTCAAAT

TTTTAGCTACCTGCTACTCTGAGTCTGCATTTCCATCTGGGGAAGAAATTCTTCAGCATG

AAGCTTCCAAAGGCTAGCTGTCACTGTGACTTAGTCTGTGAAAGGTATGCACCCATAACA

TTAGGCAAAATTAATTTCAGCCCCTTTGAACTATAACTTGTCCAAAAGACACCTCCCTGG

GTGTACGCCCAGCTCCATATGCTACATGGTGCTGGGAGCGAAGGCTGGGGGTTTGGAACT

CAGGAGCCCATCAGCCTTCTTTAGGTCCATTGTCTTTTCCAGCACACTGTATCAAATCCT

CCTGGTGACCTCACGACCCGGTGACCTCACCTTCCTTTTAACCTAGAATGTGCTGGTCCC

AAGCCAATGGCTCCCTGTCATCCTCCCTAGTGTA[A/G]CTAGTTAACAACTCTAC[G/A]TGAACTC

AAGCCAGAAATTGTGGTGCTGTCCCTTAGGGAATGGGACATATTTAA[A/G]AAAGGGTTCCCT

TTCCCCCCAGTTGTACATTTTTATCTGTGACTGGACTGGTAACTTTTTTTCATGTTATTT

TATTCAGGACCCTGGACCCCTTCCCTTATTTCCTAAACTGGTTGCATTGTTTATCTGTCC

AGTGCTAACACTGGGAAGTATTGCTTTTT[T/C]CTCTGCTCTCTGCTACTGTGTGGTATTCTG

ACAGGGGGTGGGTGTCTTTGTGTGGGTGTACTCGAAGACG[G/A]TATAGCAGCCTGTTTTCTC

TAGAATTACCATTCTAGGTTGTTTCTTGTCTCCCTCCCCCCCCCCCCCCCCCGTCTCTGC

CACTCAGATCACTTCGAGCCATCTACCACCTCTCTCATTTTGGATAATAGCCTCTCTTTT

TGCCATCGACTGCCATGTCTGATTCAGCTGCAGGAGGATTTTAAAT[T/C]AGCCAAACGTTTG

CTCACTGGGCTCACTCTGCTGCCCCAACTGATATTGATGATTGCTTCTAGAAAACAAAAA

ACAAACAAACAAACAAA[C/A][A/C]AACGCAATGGCTGGTGGTTGTACTAATACTCAGTTATTGGT

GCCTCTCTT[A/C]TACAACAGAGAGTGGAGCAGAGAGATCATCTGTGTTC[G/A]TCC[C/T]AGCTACCTTGCTGGTCAGCCATTGTACTGGTGCCCCACTCCCACTGGGAGGCTACATTCTTGCTAAGG

CAATAGAGATAAAGCAGTAAGAAGTCAAATGAAATAGTGAAAGACATGCAGTTAAATTAG

GTGACTTGCTGCTGCTGGGTTTTAACTCACTGG[T/C?]G[G/A]GAGAACAGCCTGTGTTTCTTTGAT

TCCCTCTCCTGGAGTTTGCTGGTTGGGAACTAACAGGACTGATTCTTAACATAAGAGTCT

TGCC[G/A]GCAAAGTACAAAAGGGAAAACAGAAACCCAGGAGCAGTCAGTCCCTAACAAACAT

GTACCATCCAAGCTTACAGAGGCTGAGGCAGGGGCATTGCTCTAAGTTGGATGGTATCCT

GG[A/G]TCACGTGGTAAG[C/T]TTCAAGACAAGAGGAAAGCTAGAGTTAGAGGAGATGATGGGAAA

GATCTTTCTACTGTTTCTCCAGGTTTGTCCTTAGTCTGC[A/C]GAGCTGCATCTTCTATTGCA

TGGATGGACCCTAGAAGTCCCACTCATTATTGTACCAGCTTTGAGAGTGAGGCTAGTGGT

CAGCCCAGA[T/C]TTGTTACCACAGTGGCTTCTCTTTCGTTGAGTAAGTAAACCATGAGGGTT

TCTAAAATGTTTCATTTGCTCAAGTAGAGATACACACACACATACATA[C/T]ATAT[A/G]T

[A/G][-/T][-/G]TGTGCATATATATGTGTGTATAAATATGTAGAATGTATATGTTTATGTATA

TAATCTGTCCTCAAAAACAGAAAGAAAACAAAACAAAACCTTTTTTTTTTCTGAGATGCTGTAACTACCGAGCAAAGGCTGCACATTTTGGATCAATGACCCTCCCAAGCAAAAGAGGTCCCCACAGGGGCTG

1:189778000:187821200:1

TGTATTATCATGATTGTCCTCAGCCATCACAGCCAAAATGAGATCTCCCTCTCTCTGTCT

CTTCCCTGTCTGTCTCTGTTCTCTGTGTCTCTGCTCTGTCTCTGTCTGCGTTTCTCTGTG

TGTCTCTGTGACTCTGCCTCTCCGTGTCCTCCCTCCCTCTCTCTCTCTCTTTCTCTCTCC

CTCTCCCCCTCTCTTTCTTAGTAGGTTGAGAAGCCACCAACCAGCTTCCCTAAGGCAAGC

TGTCTGCAAAGGAGCAAGCCAGCTGGCTTGGCCAAGGTTGATCAATGATGAGAGGCAGGC

TGGGTTCCAGGTCCACAACAGCCACTCAGTGGAGAGTAGGTCAGCGGCTTCCCCAAAGCC

AATTACCTGGCAGAAGTCTAGGTGCCTGGTGCTAGCCAAATTCCCTGAGCCTGATGGTGC

TGGTGGGGAGTCCATCCTGGGCTTCCTGGCAAACTGGACCAAAGGCAGCAGCAGATTGTG

CCTTCTGAGAGCCAAGGGTGGCTTTGTGGCTACCAGAACAGCGGCCGAAGGCCTGCCGCT

GCCATTCAATGTTATCCAAGTAGACTGCACATGGGGCACGGGGCACGGGACACTGTGAGC

ACTGTCCTTCAGGTGGGAGGGGCCTCGGGTGTGGAGCTGATTCACAGACTCACTCTTAAC

TTACTTGGCTGCACGGTATGAACATGAATCATGTTGTTCAGGGAGAAGGATTCAGACGCA

GTTTGGGAATCAGGAGGCATTGTCGAGACCTTTGACGGG[T/C]GTGGCAGGACTTTCACTAGT

CTAAAGGCACCTTCCCTTGTAAGTGAGATGGTATGGGAGGGAAGGCAAGACGAAGCAGAG

AGACCTTTCTGGGCAGAGACGTGAGGCTCACTTCCTTCTCCAGGGATCTCAGGATCCCAG

GCTCTTCCCTGGGACCTCCTTTCTGCTTGCTCTTCCTTCCTAACTCTGGGAATTATTTAT

CATCATCTTAGCATATCCAGACTGAAGGTGTGAGCTGAAGGGTAGAACATAGATGAATGC

TAGTTTATTTTCTGTGCCTGGACTACCTTAATGTATTGCTTCTACTCCTAACCAGGGCTT

GGTTCTAATTTTATCCATGTTCCTCAAGCCGGGTGAATGACGGCTACCCTGACCTCAGTA

TTGAGTTAGTATCAACTCATGAATAATTTGGAAGTATTTGGAAGGAAACTGGATTGTTGG

CTGATGCTTTGGCCTGTTTAAATATTTGTTCCTGCTTCCCTCACAAATGCACCCCA[T/C]TCT

TTATAATATCTAGAAGCAGGCTGAAGTGTCTCCCTCCCAGGAACTCCTTTATAGCTGTCA

ACCCAATTGATACATGTTTTGGGTCCATGTCATGACTTTTAGTCAGCATGGCTTCTATTG

GCTTACTCATCCTTCCTGGTAGCTCCGTCCCACTCAATTAATGAAGCATTAGGTTGGCAT

GAAAAGTGCATGTGACACACTGGGGAATGGTGGTTCTTCTGTTTCTAGGTTCTTGGGAAG

CTGAGGCCAAAGAGTGACTTGAGTGCAGAAATCTGAAGCCAGCCTTGGTAGCATACTGAG

ACCCTACCACAAGAAATCTGCCAAATAGAGAAAGAAAGAAACACAGAAAGAGAAGGTGGA

CTGAGTTAGATGGCTCTGTCCTCTCCGTACATGGTCCTTTTAAGTATCACCATCAACTCT

AGTTTGTGAAGTTTCCTGTTCTGGAAAATGTGAAGTTGTGGTAGAGATGGTGGCAGTGGC

TAGCACTTGCCCTGAGGAAAGCCTGGGTGTCCTCTGACGGTCCCATCTCCCATTTGTAGC

CTTCAGTCTTCCACACTAGCACTGCTAGGTTTTTGCTTTGTGAAATATATATTTCTCAAT

GATCCTGAACTTTGTATAGAACTCTGCAGAGTCATCAGGTAGTGTCTAAAGGGGGTAACA

TAGGGGGGAAAGTTATAACCTCAACTTCACTAACAGTGACAGAGTAAATGTGTTCCTGTG

TTATGT[G/A]TCTAAAGGAAGGAGGGTCTCCTCTTATCTCCCAAATGTGTCTTTGACTTTGGT

CTTTGCTTCAGCACTGAAATTCTTTCATAAGTAAAGAATTCTATTGCTACTTTAATGCCA

CTCAGAAACTGGCCTTTTCTAATGTTCCTAAGATTATGGCGCTCAGAATCCACCCCGCCC

TC[T/G]CCACTCCCACTAGCTCGCAGTGACTGAGTTTAAACTAGCAGCCCCGGTTAAGGTATT

GATTTTAACCTGAAGCCCAGAGGGACACAGGGCTGGAGTCAGCTGGACTCTTAAGCAGTC

CTGGGAGACCAGA[G/A]TCATTTGCACATCGGAAAACAGCTAATGGGCAGGGAATTAGGGCTT

AGGTAGGCCTGTCCCTTCTGT[T/C]TTCCGTCCCTCACAGCTGCTATATGTCCTGATAAAAAG

GCAGGCAGACCCAAGACACCATATTTCATTTTCTTTCTCTCTCTCTCTCTCTTAAAAGTA

AATTTTTAAGAGTCTTTTATTTCTGATAAGGACACAGGTA[T/G]CTCAGATTGGCCTCAGACC

[A/G]ACAAGAAAACTCATCCCCCATCTCTGTCTCCCAAGGGCTGGGAATACAGACATGTTGCT

TTGTTTATGTGACCCCCTAAGGGTCGAACCCAGAGCCTCATTTTTG[G/A]TTAGCGAACACTA

CTCACTGAGCTGTACCCAAGGCTGAGTATTTTT[A/T]AAGGAAACTCAAGGAAGGGAAAATGA

TAGCTAGCCATTCTTTTTCACA[T/C]TGCAAAATTCTAGT[A/T]GTAGTGAAATACTTTGCAAATA

TCAAGGCTCTAATACAAATCCTACTGGCGACATACTTGGAAGAGTATCCTCTTGTGTGTC

ATTCTGTATGGAATAGTCTTAAGTTTTTGGGCTGCCAATTACTTGTAAA[G/C]TTCTTCTTTA

AAAATGT[T/C]CTATTAGCATGTATTCTCTATACATGAT[G/-]GGTGTGTGAAGTCTTCATGCATG

GATGTGAGGTATTCTGATCACTGTTACCCCGTCACCTTCTCTTCGCCCAGGCCCGCAGCT

GCTCTTACCTCTCCCCTGCACAG[G/A][C/T]CTTGTAAGTCTCTTCCTGTTTTGGACACCCAGCTG

GGTTTCTGGCATATATCAGACGTCAGTATGAGCTGCTGTTCTGACCGGGGCAATCCCATC

CTTACCCAGTGTTTAGGACCCTAGTGGTTCCACCACACACCACCTTAAGCATCATCGTTT

GTGTTTTGTTTCACTTTGTTTGGCTCTCTGCAGTGAATGAGGGCAGTGTTCACAGCAGGC

CTGGGTTGTCTTCACATTGCTTTCTCTCCATGACTCGCTAGGGTCAGAGGTCCCCGATAA

TGCGCTGTGTGACTCCGGCCTGAGCCATTTGTGTGCCCTGGAGATCTGAGGGTAACAGGC

AGGGTCAGCATAACCTGCCAGATTCTCAGAGACCCCTCTGTGAGTTGTGGGAGGACAGTA

AAAAAGCCTGTCACGGACAGAGTAACCTTCCGTCCATGGGACTGCTTTTCATCAGATGGT

GCTCACTTTGCCCAAGTGTAACTGATGGGGTCTACAGTCAGTGCTGCACATGCTAATGTG

TTCATTTGATTCTAGCAGGACACAAACCTCAGAGGGCCACTGCAGAAGAAGGGACATTCA

CCTCTATCTAAAACATTTCACTTCACTTTCTCCCTAAAAGTTTATAGGTGTTTTGAGCTT

CTGACAACACAGGCTTAAAGATGTGTGTAGCTTGGCTCCTGTCAGTGCTTTTCTGGACTC

CTTAAGTTATTCCTCAAAGCTGTTCCTCTATTTAGGGAGGTAAAGTGTATTTCTTGTGTA

TCCTGAAAAAAAGAGGGATTTTATTTAATCCTTCCTTCTCTTTCTAGCCTATCTCC[-/T]TTTT

TCCACCTTGGAGTGGACTGCAGAGTGTGTCATCTCAGTCCTGTGTGGCCCCTAAGCCACT

TCATGCTAGTTTCATAGGGAGGAAGGGTCTCTGAGGGCTGGGAGGAAGAAGAGAAGATGA

AAGAGAAAAGAAAAGAGAGACAAAGGAAAAAAGAAATAGATGAGCCCAGTACAAATGGCC

AGGCTGTCCATTTCACTTCCAACTTCATTTCTTCCACACCCTTCTGTACAGAAAACCTTC

AAATCCCGGGGTACTGATGAG

1. Frazer KA, Eskin E, Kang HM, Bogue MA, Hinds DA, et al. (2007) A sequence-based variation map of 8.27 million SNPs in inbred mouse strains. Nature 448: 1050-1053.
